# Rational selection of ketopantoate hydroxymethyltransferase with low product feedback inhibition based on binding free energy calculation analysis

**DOI:** 10.1128/aem.00267-26

**Published:** 2026-07-10

**Authors:** Ken Zheng, Ai-Ping Pang, Fang-Ying Zhu, Yu-Hui Liu, Xiao-Jian Zhang, Qing Yang, Xiang-Yang Li, Jun-ping Zhou, Bo Zhang, Zhi-Qiang Liu, Yu-Guo Zheng

**Affiliations:** 1 State Key Laboratory of Green Chemical Synthesis and Conversion, Zhejiang University of Technology 12624, Hangzhou, Zhejiang, China; 2 The National and Local Joint Engineering Research Center for Biomanufacturing of Chiral Chemicals, Zhejiang University of Technology 12624, Hangzhou, Zhejiang, China; 3 Key Laboratory of Bioorganic Synthesis of Zhejiang Province, Zhejiang University of Technology 12624, Hangzhou, Zhejiang, China; Kyoto University, Kyoto, Japan

**Keywords:** d-PA, pantoate, ketopantoate hydroxymethyltransferase, feedback inhibition, gene mining

## Abstract

The microbial production of pantothenic acid (d-PA) is critically limited by feedback inhibition and low activity of the key enzyme ketopantoate hydroxymethyltransferase (KPHMT). To overcome this, rational enzyme mining based on computational prediction was established. Following sequence conservation analysis, molecular dynamics simulations, and binding free energy (ΔG) calculations, five representative native KPHMT enzymes were selected for experimental validation: *Ec*KPHMT from *Escherichia coli*, *Cg*KPHMT from *Corynebacterium glutamicum*, *Mp*KPHMT from *Mangrovibacter plantisponsor*, *Ep*KPHMT from *Enterovibrio pacificus*, and *Bs*KPHMT from *Bacillus subtilis. Ep*KPHMT and *Bs*KPHMT exhibited 4.25- and 4.60-fold times that of *Ec*KPHMT, respectively, with relieved pantoate feedback inhibition (IC_50_: 14.07 and 19.86 mM vs. 1.08 mM) and virtually no inhibition by d-PA. The strain expressing *Bs*KPHMT enhanced d-PA and pantoate titers by 74.30% (3.12 g/L) and 140.0% (0.84 g/L) in shake flasks, and further increased d-PA production by 55.4% in a 5 L bioreactor, over the control. This work established a predictive framework for mining superior enzymes based on *in silico* prediction, offering a valuable strategy for metabolic engineering of high-value chemicals.

**IMPORTANCE:**

The industrial-scale biosynthesis of pantothenic acid (d-PA) is often bottlenecked by the strict feedback inhibition of its key biosynthetic enzyme, ketopantoate hydroxymethyltransferase (KPHMT). This study describes a computational strategy for the mining and selection of naturally occurring KPHMTs with reduced feedback inhibition, providing superior genetic parts for metabolic applications. This approach provides a novel and rational framework for mining allostery-free enzymes, successfully delivering two highly efficient biocatalysts, *Bs*KPHMT and *Ep*KPHMT. These biocatalysts exhibit immediate potential for industrial applications, offering a direct solution to enhance the production of both pantoate and d-PA. Collectively, the integrated methodology demonstrates effective translation from fundamental discovery to practical application, presenting a generalizable model for overcoming similar metabolic bottlenecks.

## INTRODUCTION

d-Pantothenate (d-PA), also referred to as vitamin B5, is a key precursor of coenzyme A (CoA) and acyl carrier protein (ACP) (1–4). It is crucial for cellular metabolism in all organisms and thus is extensively utilized in animal feed, pharmaceutical additives, and functional foods (5–9). Traditional chemical synthesis of d-PA relies on hazardous reagents (e.g., cyanide) and multi-step reactions, generating significant toxic waste with inherent racemization issues (2, 10, 11). These processes incur high purification costs and yield pharmaceutical-incompatible products due to residual metal contaminants, fundamentally limiting industrial scalability, etc. (12). Presently, the biosynthesis routes of d-PA are highly attractive due to the advantages of simple cultivation methods, abundant raw material sources, and environmental friendliness (13–15).

**Fig 1:**
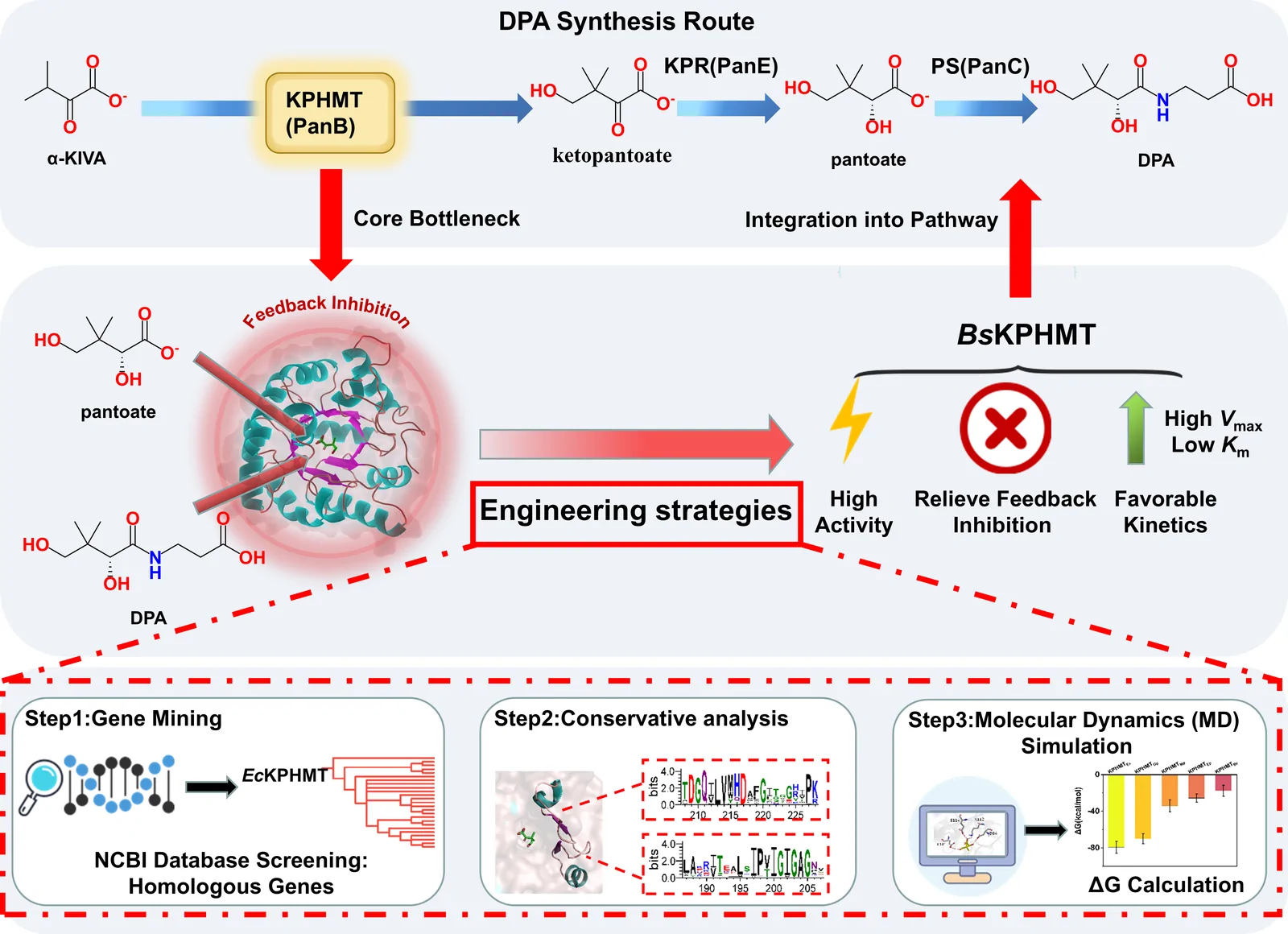
Integrated strategy for mining feedback-resistant KPHMT and their application in enhancing d-PA production. The pathway proceeds through a conserved three-enzyme cascade: ketopantoate hydroxymethyltransferase (KPHMT, EC 2.1.2.11) condenses α-ketoisovalerate and 5,10-CH_2_-THF to form ketopantoate; ketopantoate reductase (KPR, EC 1.1.1.169) reduces ketopantoate to pantoate; and pantothenate synthase (PS, EC 6.3.2.1) conjugates pantoate with β-alanine to yield d-PA.

Biosynthesis of d-PA proceeds through a conserved three-enzyme cascade. The committed step (Fig. 1), catalyzed by ketopantoate hydroxymethyltransferase (KPHMT, EC 2.1.2.11), condenses α-ketoisovalerate (α-KIVA) and 5,10-CH₂-THF to form ketopantoate (16, 17). Ketopantoate reductase (KPR, EC 1.1.1.169) then reduces ketopantoate to pantoate, which is finally conjugated with β-alanine (18) (from aspartate decarboxylase, ADC, EC 4.1.1.11) by pantothenate synthase (PS, EC 6.3.2.1) to yield d-PA (19–21). Several microbial species were investigated for d-PA biosynthesis, including native producers such as *Bacillus subtilis* (22, 23) and *Corynebacterium glutamicum* (24). However, *Escherichia coli* became a platform for d-PA biosynthesis due to its well-defined genetic system, rapid growth in low-cost media, and established industrial fermentation protocols. Through targeted modifications, researchers enhanced precursor flux and enzyme activity in *E. coli*, enabling efficient d-PA production. These engineered strains subsequently formed the basis of modern biomanufacturing processes.

The enzyme KPHMT, a rate-limiting enzyme, plays a pivotal role in the biosynthesis of d-PA (1, 18). In 1993, Jones (25) successfully cloned and overexpressed the KPHMT in *E. coli* by complementing the function of the genome library, and the same work was subsequently studied in *Aspergillus nidulans* (26), *Salmonella typhimurium* (27), *Mycobacterium tuberculosis* (28), and *B. subtilis* (22). Rubio and Downs (27) mutated the *panB* promoter to increase the expression of KPHMT, resulting in a 50% increase in coenzyme A (CoA, a derivative of d-PA) production, which highlighted that KPHMT plays a key role in the biosynthesis of d-PA. Zhang (29) constructed an *E. coli* strain DPA-9/pTrc99a -*CgpanB-CgpanC*, which replaced the promoter of key enzymes and weakened the competitive inhibition pathway, resulting in an improved accumulation of d-PA with a titer of 28.45 g/L. Site-directed mutagenesis of KPHMT from *C. glutamicum* yielded the K25A/E189S double mutant, which exhibited a 130% increase in relative activity and a decreased *K*_m_ value for α-KIVA by 0.36 mM compared to the wild type. These enhanced catalytic properties resulted in a 9.8 g/L improvement in d-PA production. Qiu obtained a double mutant V123I/K124W by modifying *Ec*KPHMT and enhanced the *de novo* synthesis pathway of 5,10-methylenetetrahydrofolate (5,10-CH_2_-THF), ultimately achieving a titer of 78.48 g/L d-PA (1). KPHMT played an important role in d-PA biosynthesis. Both metabolic engineering and protein engineering strategies of KPHMT were demonstrated to increase the fermentation titer of d-PA effectively. Meanwhile, KPHMT was subject to strong feedback inhibition by metabolic end-products such as d-PA and pantoate (13, 30). Site-directed mutagenesis by Cai et al. significantly reduced substrate inhibition in *Cg*KPHMT (30). The product d-PA exerted feedback inhibition, and ketoglutaric acid, as well as CoA, also do so (1). Despite the critical role of feedback inhibition in limiting d-PA biosynthesis, strategies directly targeting and engineering KPHMT to alleviate this allosteric constraint remain strikingly underexplored. Strategies such as promoter engineering (20) and gene dosage optimization (15) were commonly employed to enhance enzyme expression. However, these approaches primarily increased enzyme abundance without fundamentally altering the enzyme’s intrinsic catalytic properties or its sensitivity to inhibitory metabolites. Overcoming the feedback control of KPHMT continues to be a major unresolved bottleneck for enhancing industrial d-PA production.

Enzyme-directed evolution can significantly enhance the activity of enzymes and reduce inhibition. However, it is labor-intensive and lacks high-performance screening methods, which often limit its widespread use. Obtaining a suitable enzyme by mining natural enzymes is an efficient and simple method. In this study, a method combining bioinformatics and theoretical calculations was employed to obtain KPHMTs to reduce feedback inhibition. Candidate genes were initially identified through homology searches in NCBI databases. Selected enzymes were then subjected to homology modeling, MD simulations, and ΔG calculations to predict their inhibition sensitivity. Following computational screening, five representative KPHMT homologs that spanned a range of predicted inhibition sensitivity were expressed and purified for biochemical analysis. Their catalytic activities, inhibition profiles, and biochemical properties were systematically evaluated. The most promising KPHMTs were subsequently integrated into an engineered *E. coli* strain and tested under both shake-flask and 5 L bioreactor. This work not only confirms KPHMT as a critical metabolic target for d-PA production but also establishes a general strategy for mining and implementing feedback-resistant enzyme homologs in microbial cell factories.

## RESULTS AND DISCUSSION

### Mining and screening of KPHMT genes

**Fig 2:**
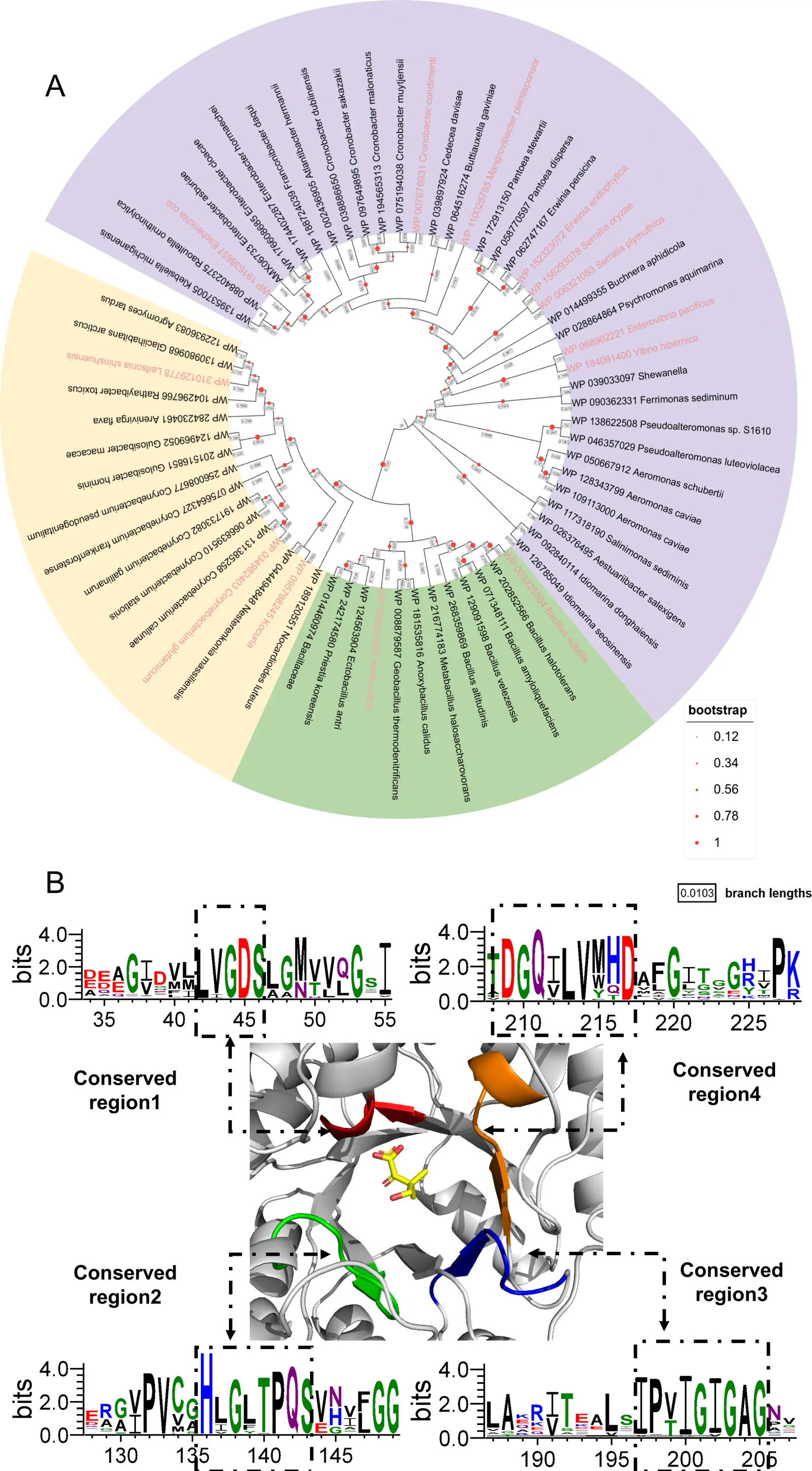
Phylogenetic analysis of KPHMTs and conserved analysis of KPHMTs. (**A**) Phylogenetic analysis of KPHMTs from diverse origins. A neighbor-joining phylogenetic tree was constructed from 65 KPHMT homologs using MEGA11 with 1,000 bootstrap replicates. The tree is divided into three major clades, highlighted in purple, green, and yellow to facilitate visual distinction of evolutionary groupings. Branch lengths corresponded to evolutionary distance, and red dots on nodes represented bootstrap values, with dot size indicating support level. (**B**) Multiple sequence conservation analysis of KPHMTs. Sequence logos depicted conservation for the amino acid regions: LVGDS (42–46), HLGLTPQS (136–143), IPVIGIGAG (197–205), and DGQILVMHD (209–217). Residues were colored by their physicochemical properties: red, acidic (D and E); blue, basic (K, R, and H); black, hydrophobic (A, V, L, I, M, F, W, and P); purple, neutral (G, S, T, Y, and C); and green, polar (N and Q).

To explore a suitable KPHMT for pantoate and d-PA biosynthesis, *Ec*KPHMT was used as a probe to search for homologous sequences in the NCBI databases. A total of 65 candidate KPHMT homologs were retrieved. Their global sequence identities relative to *Ec*KPHMT were calculated, revealing three distinct groups: 22 homologs with high similarity (>70% identity), 17 with moderate similarity (50%–70% identity), and 26 with low similarity (<50% identity). A neighbor-joining (NJ) phylogenetic tree was then constructed to assess evolutionary distances (Fig. 2A). To ensure broad coverage, we selected representatives from both closely and distantly related branches, spanning the full phylogenetic spectrum from near neighbors to far relatives. Multiple sequence alignment was performed with ESPript 3.2 (31). Four highly conserved regions around the predicted inhibitor-binding pocket were identified based on the *Ec*KPHMT crystal structure (PDB: 1M3U). These motifs are LVGDS (residues 42–46), HLGLTPQS (residues 136–143), IPVIGIGAG (residues 197–205), and DGQILVMHD (residues 209–217), respectively (Fig. 2B). The specific amino acid sequences of these four regions for all KPHMT homologs are provided in Table S1. Because amino acid differences within these functionally important regions are likely to affect catalytic or regulatory properties, we compared each homolog’s sequence within these four motifs against that of *Ec*KPHMT. For each homolog, the conserved-region similarity was scored in 25% increments: if any of the four regions differed from *Ec*KPHMT, the similarity was set to 75%; if two regions differed, 50%; if three regions differed, 25%; if all four matched perfectly, 100%. By combining the two gradients (global identity and conserved-region identity) and the phylogenetic relatedness inferred from the phylogenetic tree, 12 putative KPHMT sequences derived from *Bacillus subtilis* (*Bs*KPHMT, 43% identity to *Ec*KPHMT), *Mangrovibacter plantisponsor* (*Mp*KPHMT, 78%), *Cronobacter condimenti* (*Cc*KPHMT, 78%), *Enterovibrio pacificus* (*Ep*KPHMT, 60%), *Aeribacillus* (*Ae*KPHMT, 42%), *Kocuria* (*Ko*KPHMT, 40%), *Serratia plymuthica* (*Sp*KPHMT, 74%), *Leifsonia shinshuensis* (*Ls*KPHMT, 39%), *Vibrio hibernica* (*Vh*KPHMT, 60%), *Corynebacterium glutamicum* (*Cg*KPHMT, 39%), *Serratia oryzae* (*So*KPHMT, 74%)*,* and *Erwinia endophytica* (*Ee*KPHMT, 74%) were selected for further computational analysis and their amino acid sequences are listed in Table S2.

**Fig 3:**
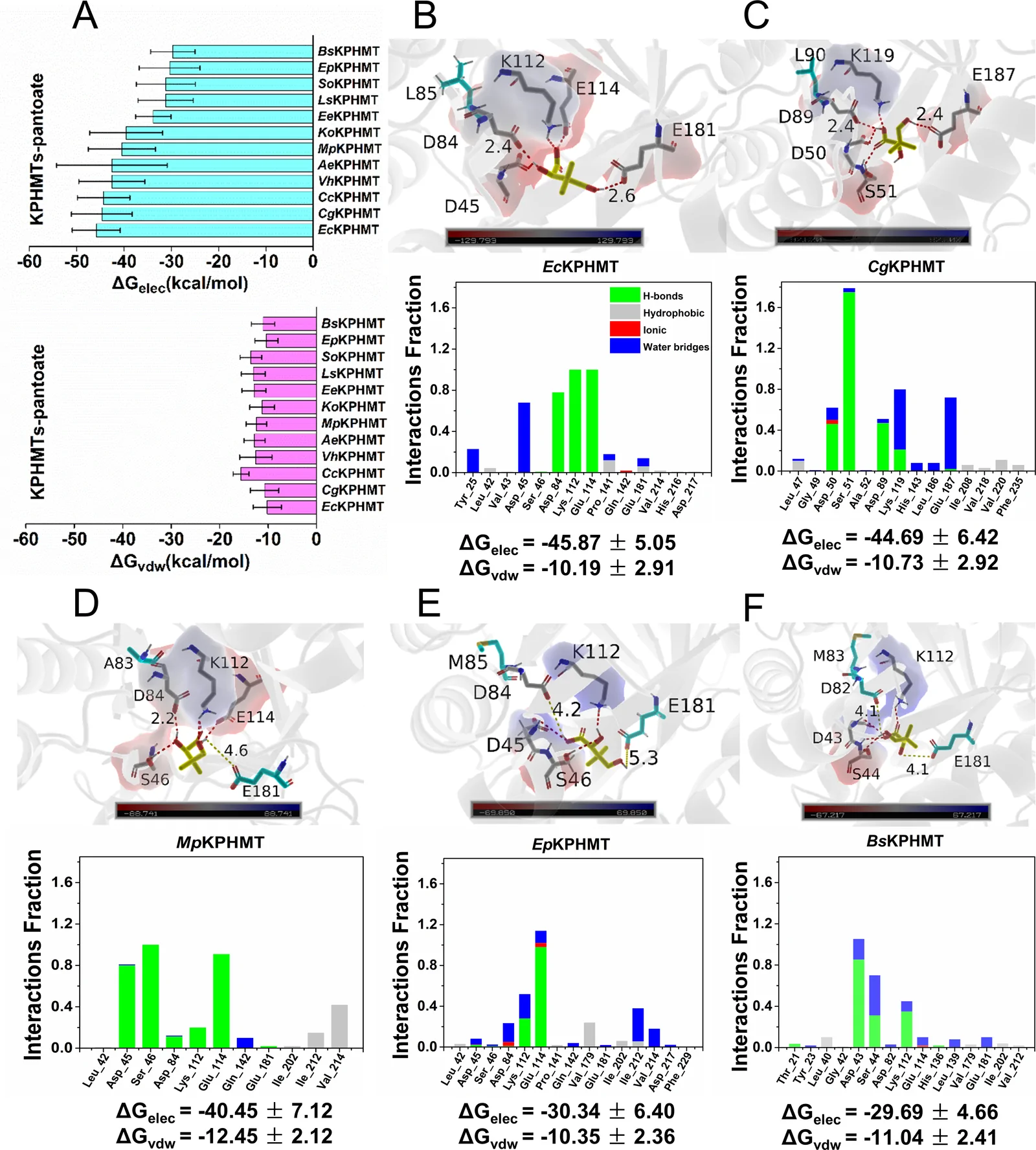
Comparative analysis of the ΔG components for pantoate binding to KPHMTs. (**A**) The electrostatic (ΔG_elec_) and van der Waals (ΔG_vdw_) components of the ΔG in the upper and lower panels, respectively. (**B through F**) Interaction fraction profiles and predicted binding poses of pantoate within the allosteric pockets of *Ec*KPHMT (**B**), *Cg*KPHMT (**C**), *Mp*KPHMT (**D**), *Ep*KPHMT (**E**), and *Bs*KPHTM (**F**). The top panel showed the binding pose of the inhibitor (stick model) within the allosteric pocket (transparent surface). Key residues were shown as sticks, and hydrogen bonds were indicated by dashed lines. The downside panel showed the fraction of specific molecular interactions (hydrogen bonds, hydrophobic interactions, ionic interactions, and water bridges) between the inhibitor and KPHMTs. The bottom data panel illustrates the ΔG between KPHMTs and the inhibitor. The three-dimensional structure of *Ec*KPHMT was obtained from the Protein Data Bank (PDB ID: 1M3U). Other structures were predicted using AlphaFold2. ΔG of pantoate with KPHMT homologs was predicted from MD simulations. Values are shown as mean ± SD calculated from snapshots taken during the equilibrium phase of the 50 ns simulation trajectory, reflecting the dynamic fluctuations of the enzyme–inhibitor interactions.

**Fig 4:**
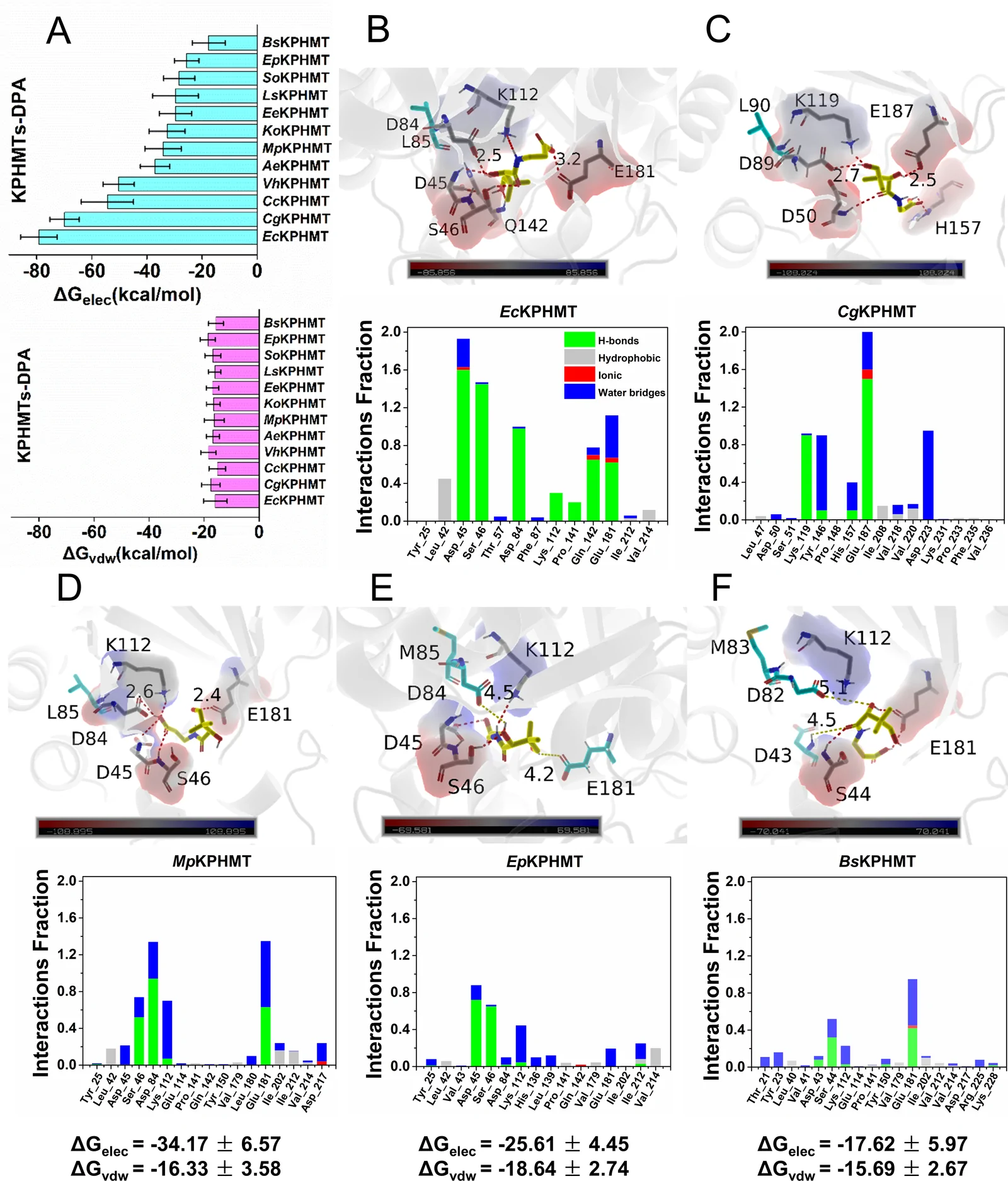
Comparative analysis of the ΔG components for d-PA binding to KPHMTs. (**A**) The electrostatic (ΔG_elec_) and van der Waals (ΔG_vdw_) components of the ΔG in the upper and lower panels, respectively. (**B through F**) Interaction fraction profiles and predicted binding poses of d-PA within the allosteric pockets of *Ec*KPHMT (**B**), *Cg*KPHMT (**C**), *Mp*KPHMT (**D**), *Ep*KPHMT (**E**), and *Bs*KPHTM (**F**). The top panel showed the binding pose of the inhibitor (stick model) within the allosteric pocket (transparent surface). Key residues were shown as sticks, and hydrogen bonds were indicated by dashed lines. The downside panel showed the fraction of specific molecular interactions (hydrogen bonds, hydrophobic interactions, ionic interactions, and water bridges) between the inhibitor and KPHMTs. The bottom data panel illustrates the ΔG between KPHMTs and the inhibitor. The three-dimensional structure of *Ec*KPHMT was obtained from the Protein Data Bank (PDB ID: 1M3U). Other structures were predicted using AlphaFold2. ΔG of d-PA with KPHMT homologs was predicted from MD simulations. Values are shown as mean ± SD calculated from snapshots taken during the equilibrium phase of the 50 ns simulation trajectory, reflecting the dynamic fluctuations of the enzyme–inhibitor interactions.

To predict the sensitivity of feedback inhibition, kinetics simulations and ΔG calculations of KPHMTs were performed to study the interaction of KPHMT with the inhibitor (pantoate and d-PA). The ΔG values provided a quantitative basis for predicting feedback-inhibiting susceptibility. Based on binding free energies calculated from MD simulations, lower ΔG values indicated stronger inhibitor binding, while higher values indicated weaker binding and potentially reduced inhibition (32). As predicted, there were significant differences in electrostatic interactions (ΔG_elec_) among various KPHMTs, while the differences in van der Waals forces (ΔG_vdw_) were relatively smaller (Fig. 3A and Fig. 4A). This indicated that the inhibitory effect is mainly due to ΔG_elec_ rather than ΔG_vdw_. Additionally, during MD simulations, the interaction fractions between the inhibitor and the KPHMTs are presented in Fig. 3B through F and Fig. 4B through F, respectively. These results suggested that hydrogen bonds and water bridges were the main factors determining the strength of the binding between the inhibitor and the KPHMTs. Based on the ΔG analysis, *Bs*KPHMT and *Ep*KPHMT emerged as the most promising candidates due to their significantly attenuated electrostatic contributions (e.g., −29.69 ± 4.66 and −30.34 ± 6.40 kcal/mol with pantoate; −17.62 ± 5.97 and −25.61 ± 4.45 kcal/mol with d-PA), indicating they are likely the least susceptible to feedback inhibition. In contrast, *Ec*KPHMT and *Cg*KPHMT displayed the values of ΔG_elec_ for the pantoate (−45.87 ± 5.05 and −44.69 ± 6.42 kcal/mol), d-PA (−79.25 ± 6.64 and −69.87 ± 5.33 kcal/mol), indicating that they were subject to strong inhibition.

For experimental validation, five enzymes were selected from the twelve candidates based on their predicted ΔG values. The two with the most negative ΔG (*Ec*KPHMT and *Cg*KPHMT) were taken as sensitive controls, and the two with the least negative ΔG (*Bs*KPHMT and *Ep*KPHMT) as resistant controls. The intermediate candidate *Mp*KPHMT was specifically chosen because its ΔG values ranked near the median of the 12 for both inhibitors (approximately 6th–7th place). This allows the intermediate to serve as an internal reference when comparing the computational ranking against experimental results.

### Enzymatic characterization and feedback inhibition profiles of selected KPHMTs

To verify the accuracy of the calculated prediction results, the five KPHMTs were expressed in *E. coli* BL21 (DE3). Under the same reaction conditions, the catalytic activities of different KPHMTs in the whole cells showed significant differences (Table 1). Among them, the activities of *Ec*KPHMT and *Cg*KPHMT were significantly lower, being 62.99 ± 1.32 and 78.74 ± 1.57 U g^−1^ DCW, respectively. In contrast, the activities of *Mp*KPHMT, *Ep*KPHMT, and *Bs*KPHMT reached 245.66 ± 3.44, 267.71 ± 2.91, and 289.75 ± 4.22 U g^−1^ DCW, respectively. Notably, *Bs*KPHMT demonstrated the highest activity, approximately 4.60 times that of the *Ec*KPHMT.

**TABLE 1:** Activities of the recombinant KPHMTs^*b*^

Recombinant strain of KPHMTs	Specific cell activity (U g_DCW_^−1^)^*a*^
*E. coli* BL21(DE3)/pET28a-*Ec*KPHMT	62.99 ± 1.32
*E. coli* BL21(DE3)/pET28a-*Cg*KPHMT	78.74 ± 1.57
*E. coli* BL21(DE3)/pET28a-*Mp*KPHMT	245.66 ± 3.44
*E. coli* BL21(DE3)/pET28a-*Ep*KPHMT	267.71 ± 2.91
*E. coli* BL21(DE3)/pET28a-*Bs*KPHMT	289.75 ± 4.22

^*a*^ DCW, dry cell weight.

^*b*^ All KPHMTs were expressed in *E. coli* BL21(DE3) harboring the pET28a vector. Enzyme activities were determined using whole cells expressing the respective KPHMT. Data are presented as the mean ± SD (*n* = 3).

**Fig 5:**
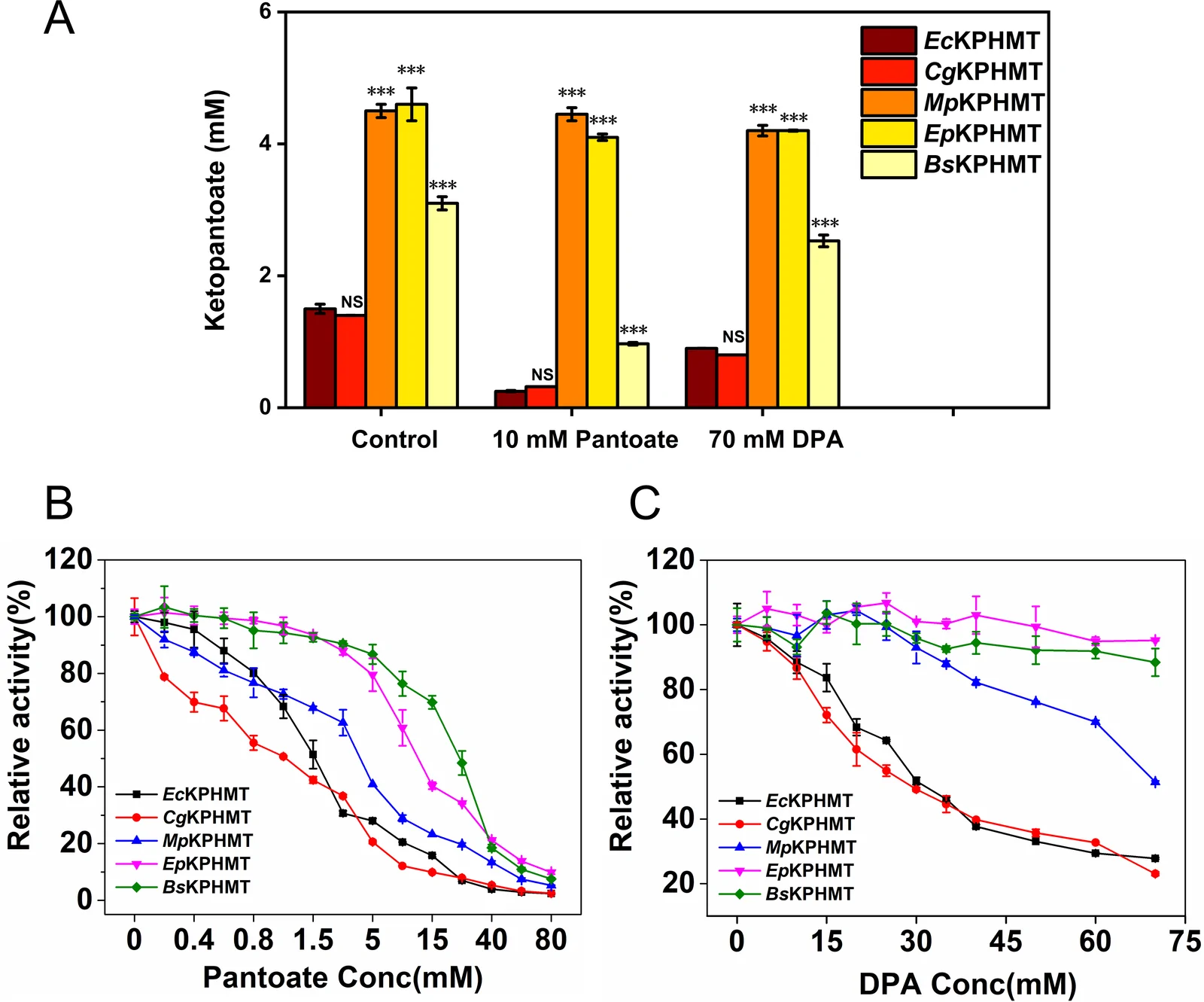
(**A**) Comparison of enzymatic activity and feedback inhibition profiles of KPHMTs under whole-cell conditions. (**B and C**) Inhibition profiles of purified KPHMT enzymes. Assays measured the inhibition of enzyme activity by pantoate (**B**) and pantothenate (**C**), respectively. All results are expressed as mean ± SD of three independent experiments. Asterisks indicate significant differences of all KPHMTs compared to the *Ec*KPHMT control group (***P < 0.001), while non-significant comparisons are labeled as NS.

To assess feedback inhibition, 10 mM pantoate and 70 mM d-PA were added to the whole-cell reaction system, respectively. In the absence of inhibitors, *Ec*KPHMT and *Cg*KPHMT produced only 1.52 ± 0.07 mM and 1.41 ± 0.01 mM ketopantoate, respectively. Their activity was severely inhibited in the presence of inhibitors, decreasing sharply to 0.25–0.32 mM in the presence of 10 mM pantoate and remaining below 0.9 mM with 70 mM d-PA. In contrast, *Bs*KPHMT and *Ep*KPHMT showed markedly higher basal activity (4.56 ± 0.10 mM and 4.67 ± 0.24 mM ketopantoate, respectively) and maintained high product levels under inhibitory conditions, with titers of 4.45 ± 0.08 mM (*Bs*KPHMT) and 4.11 ± 0.05 mM (*Ep*KPHMT) in the presence of 10 mM pantoate, and 4.24 ± 0.08 mM and 4.18 ± 0.04 mM with 70 mM d-PA (Fig. 5A). Based on these results, the KPHMTs could be clearly categorized as either inhibition-sensitive (*Ec*KPHMT and *Cg*KPHMT) or inhibition-resistant (*Bs*KPHMT and *Ep*KPHMT). The sensitive KPHMTs retained only 16.45% and 22.70% activity with 10 mM pantoate, and 57.89% and 56.03% with 70 mM d-PA. In contrast, the resistant enzymes maintained 97.59% and 88.01% activity with pantoate, and 92.98% and 89.51% with d-PA. This differential sensitivity correlated with computed ligand-binding free energies, validating the use of a low predicted binding energy as a reliable indicator of feedback resistance.

To further study the characteristics of the pure enzymes of KPHMT, five KPHMTs were purified. The protein gel electrophoresis result is shown in Fig. S3. The IC_50_ values were measured to quantify their sensitivity to the inhibitors pantoate and d-PA (Fig. 5B). *Ec*KPHMT and *Cg*KPHMT showed severe inhibition by pantoate and exhibited high sensitivity, with IC_50_ values of 1.08 mM and 1.61 mM, respectively, while *Mp*KPHMT showed moderate tolerance with an IC_50_ value of 3.49 mM. *Ep*KPHMT and *Bs*KPHMT showed extremely high tolerance, with IC_50_ values of 14.07 mM and 19.86 mM, respectively, which represented 13.03-fold and 18.39-fold increase compared to *Ec*KPHMT. *Ec*KPHMT and *Cg*KPHMT also showed severe inhibition by d-PA, with IC_50_ values of 32.56 mM and 28.70 mM, respectively, while *Mp*KPHMT exhibited moderate tolerance (IC_50_ = 72.89 mM). *Ep*KPHMT and *Bs*KPHMT, however, were nearly unaffected by d-PA (Fig. 5C).

To understand why the computational predictions (ΔG_elec_) aligned so closely with the experimental feedback inhibition profiles, a detailed structural analysis was performed at the residue level. All KPHMTs share six completely conserved acidic or basic residues within 5 Å of the inhibitor binding pocket: D46, D84, K112, E114, H136, and E181. As shown in the multiple sequence alignment (Fig. S2), these six residues are strictly conserved across all homologs and are therefore likely essential for catalysis. Therefore, the differences in feedback sensitivity cannot be explained by changes in these primary catalytic residues. Instead, the key determinants reside in more distant positions that indirectly modulate the geometry and efficiency of inhibitor binding.

Two regions were identified as critical. The first region surrounds residue D84. In the sensitive homologs *Ec*KPHMT and *Cg*KPHMT, position 85 is occupied by a small hydrophobic residue (leucine). In the resistant homologs *Ep*KPHMT and *Bs*KPHMT, this position carries a bulkier methionine. The larger side chain of methionine creates a steric hindrance that subtly pushes D84 away from the inhibitor binding pocket. This was confirmed by measuring the hydrogen bond distance between D84 and the inhibitor. In *Ec*KPHMT and *Cg*KPHMT, the D84–pantoate distance fell within the effective hydrogen bonding range. In contrast, for *Ep*KPHMT and *Bs*KPHMT, the distance increased to 4.2 Å and 4.1 Å, respectively, effectively breaking the hydrogen bond. Effective hydrogen bonds are indicated by red dashed lines, whereas ineffective ones are marked with yellow dashed lines. A similar effect was observed with d-PA, where the distances became even larger (4.5 Å and 5.1 Å in the resistant homologs). This loss of a direct hydrogen bond is not simply a matter of distance; it also disrupts the local hydrogen bond network that normally stabilizes the inhibitor. Furthermore, position 83 contributes to the steric environment around D84: *Ec*KPHMT has A83 (small, neutral), *Cg*KPHMT has V83 (slightly bulkier), *Ep*KPHMT has A83, and *Bs*KPHMT has T83 (polar, with a hydroxyl group). The threonine in *Bs*KPHMT may introduce an additional water-mediated interaction that further reorients D84.

The second region involves residue E181. Variations at positions 176, 177, and 185 alter the spatial arrangement of the loop carrying E181. In sensitive homologs, E181 is positioned optimally to form a hydrogen bond with the inhibitor’s hydroxyl groups. In resistant homologs, the loop geometry is slightly displaced, increasing the distance between E181 and the inhibitor. As a result, the number of polar contacts between the enzyme and the inhibitor is reduced. This reduction directly lowers the local electrostatic potential around the binding pocket, which was confirmed by the computed ΔG_elec_ values. For pantoate, *Ec*KPHMT and *Cg*KPHMT gave strongly negative ΔG_elec_ values (–45.87  ±  5.05 and –44.69  ±  6.42 kcal/mol), indicating strong electrostatic attraction. *Ep*KPHMT and *Bs*KPHMT, however, showed much less negative values (–30.34  ±  6.40 and –29.69  ±  4.66 kcal/mol), reflecting a substantial loss of electrostatic stabilization. For d-PA, the difference was even more striking: sensitive homologs gave –79.25  ±  6.64 and –69.87  ±  5.33 kcal/mol, while resistant ones gave only –25.61  ±  4.45 and –17.62  ±  5.97 kcal/mol. The more pronounced effect for d-PA likely arises from its larger size and additional polar groups, which amplify the consequences of losing specific hydrogen bonds.

In summary, the mechanistic basis for feedback inhibition relief in *Bs*KPHMT and *Ep*KPHMT is multifactorial. It involves subtle amino acid substitutions in the vicinity of conserved catalytic residues (positions 83, 85, 176, 177, and 185). These substitutions alter the distance and geometry of key hydrogen bonds (D84 and E181) to the inhibitor, thereby reducing the number of polar interactions. The consequent decrease in ΔG_elec_ was accurately captured by the MD simulations. For pantoate, computed ΔG_elec_ values for *Ec*KPHMT and *Cg*KPHMT were –45.87 and –44.69 kcal/mol, with IC_50_ values of 1.08 mM and 1.61 mM, indicating strong binding and high sensitivity. In contrast, *Ep*KPHMT and *Bs*KPHMT had less negative ΔG_elec_ (–30.34 and –29.69 kcal/mol) and much higher IC_50_ (14.07 mM and 19.86 mM), representing 13- and 18-fold greater tolerance. For d-PA, the trend was more pronounced: sensitive homologs showed ΔG_elec_ of –70 to –80 kcal/mol and IC_50_ of 28–33 mM, while resistant homologs had ΔG_elec_ of –17 to –25 kcal/mol and IC_50_ not reachable. The strong agreement between the computed ΔG_elec_ and the experimental IC_50_ values validates the computational screening strategy and provides a clear structural rationale for the observed functional differences.

Notably, the inhibition observed in whole-cell catalysis assays was less pronounced than observed with purified enzymes. This attenuated inhibitory effect was largely attributed to the hindered mass transfer across the cell membrane (33), which limited intracellular substrate accumulation and altered the apparent inhibition kinetics *in vivo*.

### Characterization of KPHMT enzymes from different species

Enzymatic properties of the purified KPHMTs were systematically characterized. The substrate 5,10-CH_2_-THF was generated *in situ* using 1 mM formaldehyde and 0.5 mM tetrahydrofolate intentionally kept below saturation levels to avoid interference with kinetic measurements. Assays were performed with α-KIVA concentrations ranging from 0 to 40 mM. *Ec*KPHMT and *Cg*KPHMT exhibited similar catalytic efficiencies, with *V*_max_ values of 7.85 ± 0.20 and 8.02 ± 0.38 U/mg, respectively. In contrast, *Mp*KPHMT, *Ep*KPHMT, and *Bs*KPHMT showed significantly higher *V*_max_ values of 13.10 ± 0.18, 17.87 ± 0.48, and 18.98 ± 0.72 U/mg, corresponding to 1.67-, 2.28-, and 2.42-fold increases over *Ec*KPHMT (Table 2). The *K*_m_ values for *Ec*KPHMT, *Cg*KPHMT, *Mp*KPHMT, *Ep*KPHMT, and *Bs*KPHMT were 4.0 ± 0.21, 4.40 ± 0.32, 1.20 ± 0.06, 2.92 ± 0.21, and 3.22 ± 0.35 mM, respectively. Among them, *Mp*KPHMT showed the lowest *K*_m_ value, indicating that it exhibited the highest substrate affinity.

**TABLE 2:** Comparison of KPHMT kinetic parameter data^*a*^

Enzyme	*V*_max_ (U/mg^−1^)	*K*_m_ (mM)	*K*_cat_ (s^−1^)	*K*_cat_/*K*_m_ (s^−1^*mg^−1^)
*Ec*KPHMT	7.85 ± 0.20	4.0 ± 0.21	3.76 ± 0.10	0.94 ± 0.03
*Cg*KPHMT	8.02 ± 0.38	4.40 ± 0.32	3.88 ± 0.19	0.88 ± 0.04
*Mp*KPHMT	13.10 ± 0.18	1.20 ± 0.06	6.33 ± 0.09	5.28 ± 0.08
*Ep*KPHMT	17.87 ± 0.48	2.92 ± 0.21	8.70 ± 0.32	2.97 ± 0.11
*Bs*KPHMT	18.98 ± 0.72	3.22 ± 0.35	9.70 ± 0.46	3.01 ± 0.14

^*a*^ Kinetic parameters were determined in 2 mL EP tubes, where 1 mL reaction system contained phosphate buffer (200 mM, pH 7.0), 2 mM MgCl_2_, 1 mM formaldehyde, 0.5 mM THF, 0.8–40 mM α-KIVA, and 10 μg of purified KPHMTs. All experiments were repeated three times.

**Fig 6:**
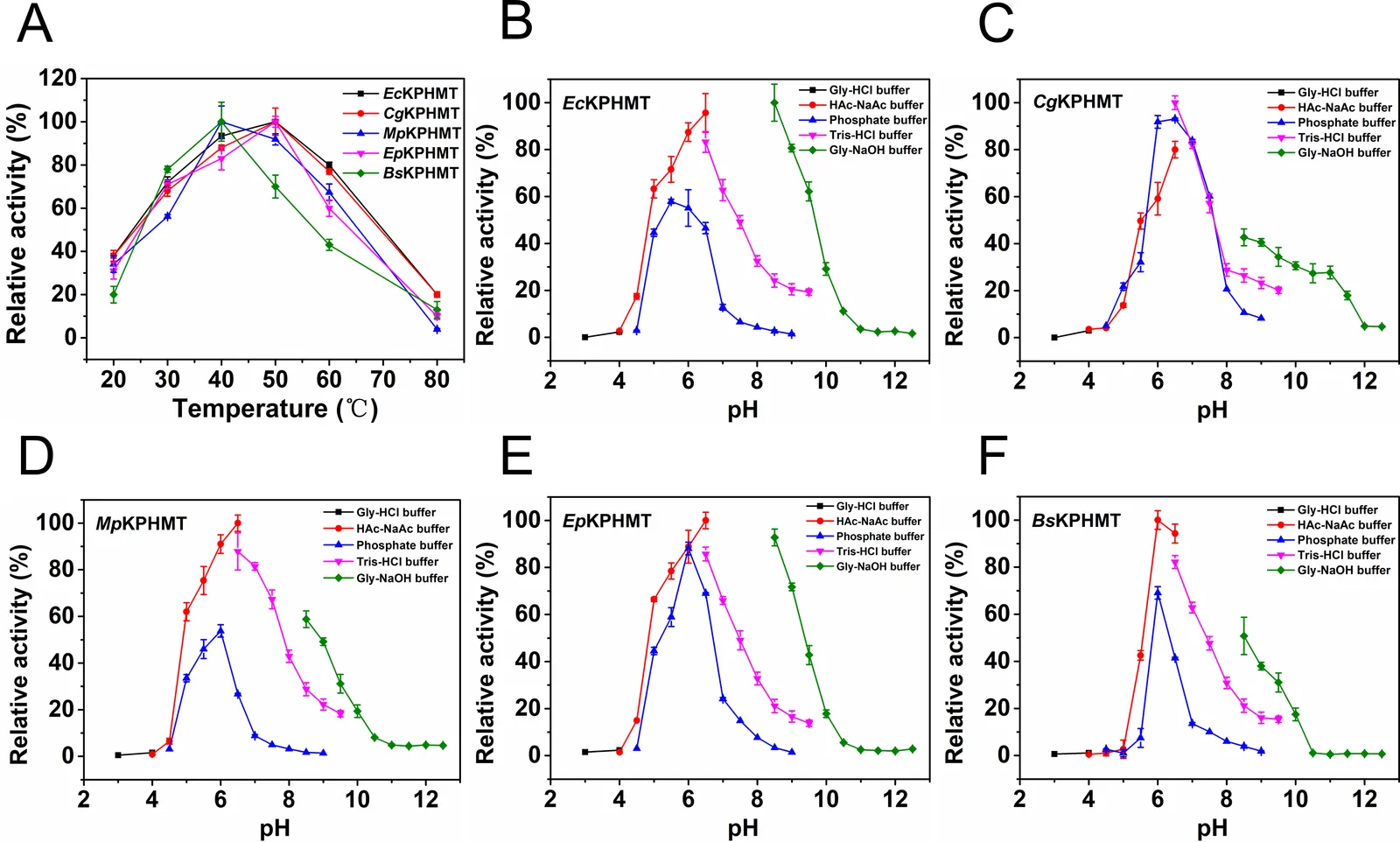
Optimal temperature and pH profiles of the five KPHMTs. (**A**) represented the optimal temperature of KPHMTs. (**B through F**) correspond to *Ec*KPHMT, *Cg*KPHMT, *Mp*KPHMT, *Ep*KPHMT, and *Bs*KPHMT, respectively. The enzymatic activity of KPHMT homologs was measured across a range of temperatures to determine their respective optimal reaction conditions. Enzymatic activities were measured under standard assay conditions and are presented as the relative activity, with the maximum value for each enzyme set to 100%.

The effects of temperature and pH on enzymatic activity were subsequently evaluated. All KPHMTs showed optimal activity between 40°C and 50°C (Fig. 6A). Citrate buffer was excluded as it inhibited KPHMT activity (Fig. S4). However, at 30°C, which was the optimal temperature for d-PA fermentation, the enzyme activity of KPHMTs decreased by approximately 20%–50% compared to the optimum. The enzymatic activity of *Mp*KPHMT decreased most significantly, reaching only 56.73% of that at the optimal temperature, whereas *Bs*KPHMT was 79.25%. Despite *Mp*KPHMT exhibiting the lowest *K*_m_ value, indicating its highest affinity for the substrate α-KIVA. Due to the thermal stability of *Mp*KPHMT being severely limited, at the optimal fermentation temperature of 30°C, its activity decreases significantly, resulting in its less favorable application in the fermentation of pantoate and d-PA compared to *Ep*KPHMT and *Bs*KPHMT.

The pH profile of each enzyme was determined using a series of buffers covering pH 3.0–12.5. All enzymes exhibited maximum activity between pH 6 and 7 (Fig. 6B through F). Notably, since the pre-synthesized 5,10-CH₂-THF substrate solution is weakly acidic (pH 4–5), data obtained under alkaline conditions may slightly overestimate the true values.

Divalent metal ions are essential for the catalytic function of KPHMT. The crystal structure of *E. coli* KPHMT (PDB: 1M3U) shows a Mg^2+^ ion coordinated in the active site, where it participates in substrate (α-KIVA) binding and stabilizes the transition state during the hydroxymethyl transfer reaction (34). Consistent with this known requirement, we systematically evaluated the effects of various metal ions on the activity of the selected KPHMT homologs. As shown in Table 3, KPHMT activity was strongly dependent on divalent metal ions. Among the ions tested, Co^2+^ and Mg^2+^ markedly enhanced catalysis, with *Ep*KPHMT showing the most pronounced activation, exhibiting 218-fold and 230-fold increases in activity, respectively. Other metal ions, including all monovalent and trivalent metal ions, showed minimal effects on enzyme activity. These findings confirm that KPHMT is a divalent metal-dependent enzyme. The strong activation by Co^2+^, which in some cases exceeded that of Mg^2+^, was unexpected. While Mg^2+^ is the presumed physiological cofactor due to its abundance in the cellular environment, Co^2+^ can serve as an effective substitute *in vitro*. This phenomenon likely reflects the plasticity of the metal-binding site. Compared with Mg^2+^, Co^2+^ has a different coordination geometry primarily due to its larger ionic radius and distinct electronic configuration. Moreover, Co^2+^ has a higher affinity for nitrogen and sulfur ligands compared to Mg^2+^, which further diversifies its possible coordination environments. Our results not only confirm the divalent metal dependence but also reveal that the extent of activation varies among homologs, suggesting subtle differences in the metal-binding pocket. These findings may guide future engineering of the metal-binding site to further enhance KPHMT catalytic efficiency.

**TABLE 3:** Effects of metal ions and EDTA on the activities of KPHMTs

Metal ion (2 mM)	Relative activity (%)				
	*Ec*KPHMT	*Cg*KPHMT	*Bs*KPHMT	*Mp*KPHMT	*Ep*KPHMT
Control	100	100	100	100	100
Na^+^	112.21 ± 1.23	118.05 ± 3.24	89.94 ± 3.87	88.86 ± 0.64	105.61 ± 3.28
K^+^	88.06 ± 0.93	76.54 ± 0.88	125.65 ± 7.63	123.10 ± 4.51	83.94 ± 1.66
Ni^2+^	795.55 ± 11.13	636.45 ± 3.27	1,147.08 ± 23.07	2,504.0 ± 25.67	13,983.91 ± 118.63
Cu^2+^	111.42 ± 2.37	89.67 ± 1.19	96.47 ± 1.69	84.60 ± 2.75	106.34 ± 5.20
Ca^2+^	127.38 ± 1.82	112.68 ± 5.08	101.82 ± 0.74	92.34 ± 3.36	118.87 ± 4.93
Sn^2+^	218.20 ± 8.27	148.61 ± 4.47	92.76 ± 1.99	92.39 ± 0.69	100.0 ± 0.90
Fe^2+^	118.04 ± 2.68	93.27 ± 1.07	940.23 ± 17.87	260.23 ± 5.27	854.81 ± 12.84
Co^2+^	1,931.82 ± 43.44	2,312.33 ± 73.81	22,540.50 ± 521.45	7,760.0 ± 89.50	23,032.32 ± 424.72
Mg^2+^	758.15 ± 21.42	563.41 ± 19.93	20,144.25 ± 628.28	6,720.0 ± 71.93	21,806.51 ± 246.97
Mn^2+^	522.79 ± 9.68	622.58 ± 8.89	17,500.0 ± 353.50	2,620.0 ± 20.50	19,306.52 ± 56.40
Zn^2+^	130.06 ± 1.64	165.31 ± 4.46	511.84 ± 11.25	184.61 ± 2.27	1,435.51 ± 45.59
Al^3+^	90.93 ± 0.98	81.17 ± 1.25	90.97 ± 0.94	84.63 ± 1.87	143.87 ± 4.88
Ru^3+^	106.81 ± 2.40	123.35 ± 5.30	154.51 ± 1.88	84.63 ± 1.81	93.81 ± 1.22
Fe^3+^	134.15 ± 3.86	132.84 ± 1.20	112.74 ± 1.69	123.18 ± 0	256.30 ± 15.17
EDTA	88.01 ± 0.99	82.51 ± 0.81	76.55 ± 0	90.0 ± 2.24	83.91 ± 1.98

### Fermentation performance of strains expressing distinct KPHMTs in a shake flask

**Fig 7:**
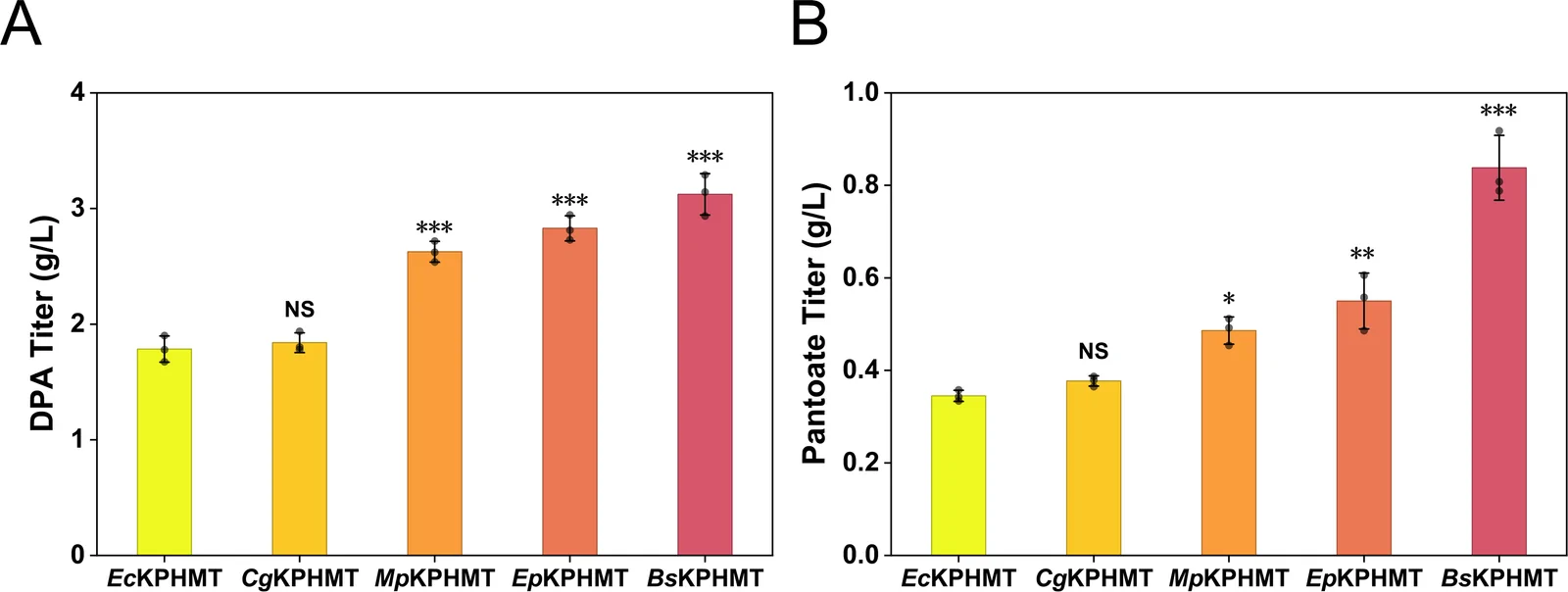
The feedback inhibition of KPHMTs by d-PA (**A**) and pantoate (**B**). Values are shown as mean ± SD (*n* = 3). Asterisks indicate significant differences between all KPHMTs compared to the *Ec*KPHMT control group. Asterisks denote statistical significance as follows: *P < 0.05, **P < 0.01, ***P < 0.001; NS, not significant (*P* ≥ 0.05).

To assess the effect of different KPHMT on d-PA biosynthesis, the plasmid pTrc99a-*panB-CgpanC* with each KPHMT was ligated (Table S2) and introduced in *E. coli* DPA11A (35). As anticipated, strains expressing the inhibition-resistant *Ep*KPHMT and *Bs*KPHMT showed superior d-PA titers in shake flask fermentations. The control strain expressing *Ec*KPHMT produced 1.79 ± 0.09 g/L d-PA, while the *Cg*KPHMT achieved a similar titer of 1.84 ± 0.07 g/L. In contrast, strains expressing *Ep*KPHMT and *Bs*KPHMT achieved titers of 2.83 ± 0.09 g/L and 3.12 ± 0.15 g/L, representing an enhancement of 58.1% and 74.3%, respectively (Fig. 7A). These results confirm the critical role of KPHMT in d-PA biosynthesis.

The effect of each KPHMT on pantoate production was also assessed by expressing KPHMT in *E. coli* DPA11A/∆*panC*. 50 μM sodium pantothenate was supplemented to sustain growth. Pantoate titers reached 0.35 ± 0.01 g/L (*Ec*KPHMT), 0.38 ± 0.01 g/L (*Cg*KPHMT), 0.49 ± 0.02 g/L (*Mp*KPHMT), 0.55 ± 0.03 g/L (*Ep*KPHMT), and 0.84 ± 0.04 g/L (*Bs*KPHMT) (Fig. 7B). The 140.0% increase in pantoate achieved using *Bs*KPHMT significantly exceeded the increase achieved by d-PA, indicating that KPHMT is a major bottleneck in the synthesis process of pantoate.

### Fed-batch fermentation in a 5 L fermenter

**Fig 8:**
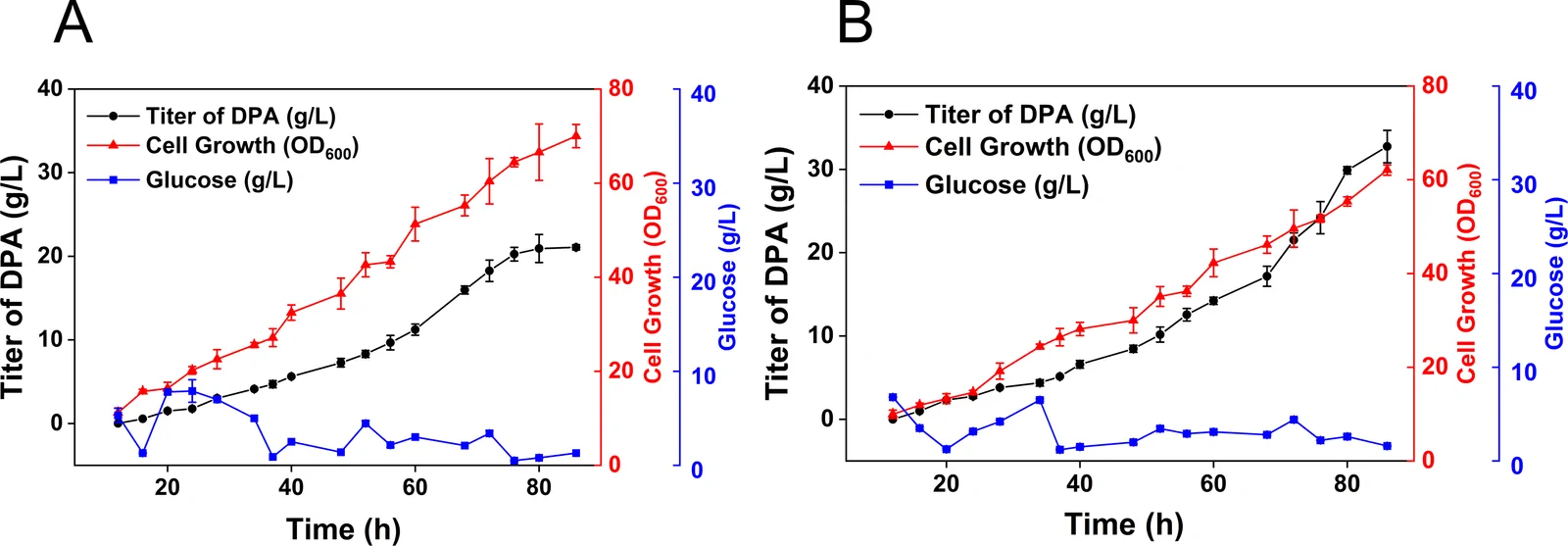
The fermentation performance of strains expressing *Ec*KPHMT (**A**) and *Bs*KPHMT (**B**) in a 5 L bioreactor under fed-batch conditions. d-PA titer, cell growth, and glucose concentration were monitored throughout the process.

To further explore the industrial potential of the feedback-resistant *Bs*KPHMT in d-PA production, fed-batch fermentation was conducted in a 5 L bioreactor. After 86 h of fermentation, the strain carrying *Bs*KPHMT produced 32.74  ±  1.95  g/L d-PA (Fig. 8), which was a 55.40% increase compared to the strain expressing *Ec*KPHMT (21.07  ±  0.32  g/L d-PA). The yield of the *Bs*KPHMT strain was higher, but its final cell density was lower, with an OD_600_ at 62.10  ±  1.10. In contrast, the OD_600_ of the *Ec*KPHMT strain reached 70.10  ±  2.47. This indicated that the increase in the concentration of d-PA synthesis was not caused by an increase in cell concentration, but rather that more carbon fluxes were directed toward d-PA synthesis. This means that alleviating feedback inhibition can effectively guide the metabolic flow toward product synthesis.

A direct comparison with the recent study by Qiu et al. is warranted to contextualize our findings. Qiu et al. achieved an impressive d-PA titer of 78.48 g/L in a 5 L bioreactor by engineering KPHMT and a multi-module engineering strategy. The V123I/K124W double mutant of KPHMT was reported to enhance intersubunit interactions through the formation of an aromatic cage involving W124, leading to improved enzyme stability and a 2.5-fold increase in d-PA production compared to the wild-type KPHMT. However, the effect of these mutations on feedback inhibition by pantoate or d-PA was not reported. In contrast, our work focuses specifically on alleviating feedback inhibition at the enzyme level without any pathway engineering. The feedback-resistant *Bs*KPHMT identified in our study exhibited an IC_50_ for pantoate of 19.86 mM (vs. 1.08 mM for *Ec*KPHMT) and virtually no inhibition by d-PA, properties that have not been demonstrated for the Qiu mutant. Sequence analysis reveals that our natural homologs carry substitutions in the allosteric pocket (methionine instead of leucine at position 85, and differences at positions 176, 177, and 185 in *Bs*KPHMT) that reduce electrostatic binding affinity (ΔG_elec_ = −29.69 vs. −45.87 kcal/mol for *Ec*KPHMT), a distinct mechanism from the intersubunit stabilization proposed for the V123I/K124W mutant. These two approaches are complementary. For industrial applications requiring rapid strain development or modular genetic modification, our feedback-resistant homologs provide a “drop-in” solution that immediately confers resistance without extensive pathway engineering. For applications where maximum absolute titer is the primary goal, Qiu’s multi-module engineering strategy achieves higher production. Importantly, because the mutations are in different regions of the enzyme (loop vs. allosteric pocket), combining them could potentially yield an enzyme with both high stability/activity (from Qiu’s mutations) and low feedback inhibition (from our natural homologs). Our computational screening pipeline provides a generalizable strategy to identify such naturally evolved solutions that can be combined with rational engineering for further improvement.

The pronounced functional dichotomy between inhibition-sensitive and resistant KPHMTs provides important mechanistic insights. The resistance of *Bs*KPHMT and *Ep*KPHMT stems primarily from reduced electrostatic binding affinity at the inhibitor binding site, rather than from major structural alterations or catalytic impairment. Detailed structural analysis revealed that sensitive homologs maintain effective hydrogen bonds between key residues (D84 and E181) and the inhibitor. In resistant homologs, substitutions at nearby positions (methionine instead of leucine at position 85, and differences at positions 176, 177, and 185) increase the distance of these residues from the inhibitor, breaking the hydrogen bonds and reducing the number of polar contacts. This results in less favorable ΔG_elec_ values and weaker feedback inhibition, while catalytic efficiency remains unchanged. The integrated approach successfully linked computational predictions with experimental outcomes, demonstrating that rational enzyme mining can effectively identify biocatalysts with optimized kinetic properties.

### Conclusions

This study established a rational enzyme mining strategy to alleviate the severe feedback inhibition of the key rate-limiting enzyme KPHMT through computational guidance. Through genomic screening, molecular dynamics simulations, and binding free energy calculations, the naturally occurring KPHMT homologs (*Bs*KPHMT and *Ep*KPHMT) with dramatically reduced feedback inhibition and high catalytic activity were successfully identified. Fermentation validation confirmed that these homologs significantly improved d-PA and pantoate production. The successful identification and implementation of feedback-resistant KPHMTs not only advanced our understanding of allosteric regulation in metabolic enzymes but also provided readily applicable genetic tools for improving the industrial production of d-PA and related compounds.

## MATERIALS AND METHODS

### Bacterial strains, vectors, medium and chemicals

All the strains and vectors are listed in Table S2. The vector pET-28a (New England Biolabs, Hitchin, UK) was selected for constructing and expressing the recombinant KPHMTs protein. The plasmid pTrc99a-*panB-CgpanC* (12) was used for the synthesis of pantoate and d-PA. The gene of *Ec*KPHMT was cloned from the genome of *E. coli* W3110, and others were mined in the NCBI database and synthesized by Tsingke Biotech (Hangzhou, China) after codon optimization. The gene of PS was cloned from *C. glutamicum* ATCC 13032, which has the highest values ever reported (36).

The microbial cultivation was performed using Luria-Bertani (LB) broth, comprising 5 g/L yeast extract, 10 g/L peptone, and 10 g/L NaCl. To achieve an optimal pH level for growth, the initial medium pH was set to 7.5 by the addition of a 1 M NaOH solution. LB broth was supplemented with suitable antibiotics, including kanamycin (Kan) (Sango Biotech, Shanghai, China). The expression of KPHMTs and PS was induced by 0.1 mM isopropylthio-β-galactoside (IPTG) from Sango Biotech in Shanghai, China. The MS medium was employed for the biosynthesis of both d-PA and pantoate, which contained 16 g/L (NH_4_)_2_SO_4_, 20 g/L glucose, 4 g/L yeast extract, 2 g/L KH_2_PO_4_, 0.5 g/L MgSO_4_, and 1 mL/L metal salt solution, was supplemented with 10 g/L sterilized CaCO_3_ for the biosynthesis of pantoate and d-PA in a shake flask. The metal salt solution consisted of 10 g/L FeSO_4_·7H_2_O, 10 g/L ZnSO_4_·7H_2_O, 10 g/L CaCl_2_, 0.2 g/L CuSO_4_, and 0.02 g/L NiCl_2_·7H_2_O. 5 mg/L VB_1_, 2 mg/L VB_12_, 2 g/L β-alanine, 0.4 g/L isoleucine, 50 mg/L Kan, and 48 mg/L IPTG were added when strains cultured in shake flask.

THF (Cas: 135-16-0), ketopantolactone (KPL, Cas: 13031-04-4), d-pantolactone (Cas: 599-04-2), and sodium d-pantothenate (Cas: 867-81-2) were purchased from Aladdin Scientific Ltd (Shanghai, China). α-KIVA (Cas: 759-05-7) was purchased from Meryer Chemical Technology Ltd (Shanghai, China). The remaining chemicals were obtained commercially and were of analytical grade.

### Gene mining and phylogenetic analysis

Homologous sequences of KPHMT were retrieved from the NCBI database using the *Ec*KPHMT sequence (NCBI ID: WP_191638657) as a query. Multiple sequence alignment was performed using Clustal W, and the resulting alignment was used for phylogenetic analysis. Based on the resulting alignment, sequence conservation was analyzed and visualized using ESPript 3.2 (31). The NJ method (37) was employed to analyze the phylogenetic relationships among KPHMT enzymes. A bootstrap analysis with 2,000 replications was performed using MEGA11 software (38). Branch length annotations were added to the phylogenetic tree to indicate evolutionary distances, which were calculated using the Poisson correction method and expressed as amino acid substitutions per site. Red dots on the tree represent bootstrap values, with varying sizes indicating their strength. A total of 65 amino acid sequences were included in this analysis after removing all ambiguous positions between sequence pairs through a pairwise deletion option.

### MD simulations

All MD simulations were performed using the GROMACS 2025 software package. The three-dimensional structure of *Ec*KPHMT was obtained from the Protein Data Bank (PDB ID: 1M3U). For the remaining KPHMT homologs (*Bs*KPHMT, *Cg*KPHMT, *Mp*KPHMT, *Ep*KPHMT, etc.), their structures were predicted using AlphaFold2, and for each homolog, the model with the highest predicted local distance difference test (pLDDT) score was selected. The protein was modeled using the Amber99SB-ILDN force field, while the topologies and coordinate files for the ligand (pantoate or d-PA) and cofactor (Mg^2+^) were generated using AmberTools24 with the General Amber Force Field 2 (GAFF2). The Amber-format files were subsequently converted to GROMACS format using the acpype.py script. The complex was solvated within a rhombic dodecahedron box of TIP3P water molecules, ensuring a minimum distance of 10 Å from the protein surface to the box boundaries. The system was neutralized by adding Na^+^ ions to the solvent. Energy minimization was conducted using the steepest descent method (5,000 steps) to remove steric clashes. Subsequently, the system underwent two-stage equilibration for 100 ps each: an NVT ensemble (constant number of particles, volume, and temperature) and an NPT ensemble (constant number of particles, pressure, and temperature) to reach the desired state. Finally, a 50 ns production MD run was executed. The simulation trajectories were analyzed using the built-in tools of GROMACS 2025, and results were visualized and further processed using DuIvyTools (v 0.3.0) and PyMOL.

### Construction of recombinant strains

For protein overexpression and purification, each *panB* gene was cloned into the vector pET-28a (Table S1) and transformed into *E. coli* BL21(DE3). To evaluate the impact on the biosynthesis of pantoate, the corresponding *panB* gene was ligated to pTrc99a and transformed into our previously reported *E. coli* strain DPA11A/∆*panC* (35). For d-PA production, the *panB* gene was assembled together with the *panC* gene into pTrc99a, and the resulting plasmid was introduced into our previously reported *E. coli* DPA11A (35).

### Protein expression and purification

Protein expression was induced with 0.1 mM IPTG at an OD_600_ of 0.6–0.8, followed by incubation at 28 °C for 12 h. Cells were harvested by centrifugation, resuspended in lysis buffer (100 mM PBS, pH 7.5), and disrupted by sonication on ice. The crude lysate was loaded onto a Ni-NTA column pre-equilibrated with binding buffer (20 mM NaH_2_PO_4_, 300 mM NaCl, pH 7.5). After washing with binding buffer containing 50 mM imidazole, the target protein was eluted with the same buffer supplemented with 500 mM imidazole. The eluted protein was dialyzed against 20 mM PBS (pH 7.5). The molecular weight and protein purity were verified by SDS-PAGE, and concentration was determined using a bicinchoninic acid (BCA) assay kit.

### Enzymatic activity assay

The substrate 5,10-CH_2_-THF, which serves as the one-carbon donor in the KPHMT-catalyzed reaction, is commercially inaccessible and chemically labile. It was therefore generated *in situ* via a spontaneous condensation of formaldehyde and THF at room temperature.

The assay was performed in a 1 mL reaction system containing cell catalyst (15 g/L wet cell weight) or purified enzyme (10 μg/mL) and 2 mM MgCl_2_ or CoCl_2_ in 100 mM phosphate buffer (pH 7.5). The substrate solution was prepared separately by incubating 3 mM α-KIVA, 15 mM THF, and 9 mM formaldehyde in the phosphate buffer at 37 °C for 10 min with shaking (600 rpm). Then, 10 g/L cell or 10 μg/L enzyme was added to the substrate solution and reacted at 37 °C for 12 min with shaking. The reaction was terminated by adding 150 μL of 6 M HCl, followed by incubation for 30 min to allow acid-catalyzed lactonization of ketopantoate to KPL. Subsequently, KPL was subjected to triple extraction with an equal volume of ethyl acetate and quantified by gas chromatography (GC). One unit of enzyme activity was defined as the amount of enzyme required to produce 1 μmol KPL per minute under the assay conditions. A schematic of the complete assay procedure is provided in Fig. S1.

### Feedback inhibition of pantoate and d**-**PA on KPHMT

Early studies by Powers and Snell demonstrated that KPHMT activity is subject to feedback inhibition by pathway metabolites, pantoate and d-PA. To evaluate whether this inhibition is conserved across KPHMTs from diverse origins, the activities of each purified KPHMT were assayed in the presence of increasing concentrations of either inhibitor. The assay conditions were identical to the standard activity measurement, except for adding pantoate or d-PA to the reaction mixture. The residual activity at each inhibitor’s concentration was determined to quantify the inhibition sensitivity of each KPHMT.

### Effects of temperature and pH on KPHMT

The optimal temperature for assessing the activity of KPHMT was evaluated across a range of temperatures from 20°C to 80°C. Meanwhile, the optimum pH was identified by measuring activity at 37 °C using a series of overlapping buffers: Gly-HCl (pH 2.2–5.0), HAc-NaAc (pH 3.6–5.8), phosphate (pH 5.8–8.0), Tris-HCl (pH 7.1–9.0), and Gly-NaOH (pH 8.6–10.6).

### Effects of metal ions on KPHMT

Based on prior reports that metal ions enhance KPHMT catalysis, 14 different metal ions (K^+^, Na^+^, Ni^2+^, Cu^2+^, Ca^2+^, Sn^2+^, Fe^2+^, Co^2+^, Mg^2+^, Mn^2+^, Zn^2+^, Ru^3+^, Al^3+^, and Fe^3+^) with a final concentration of 2 mM were added. The reaction was incubated at 37°C for 30 min, and the enzyme activity was determined.

### Culture conditions for the biosynthesis of pantoate and d-PA

To compare the titer of d-PA in strains expressing different KPHMTs, a single colony was inoculated in 10 mL LB medium containing 50 mg/L Kan as seeds and cultured at 37°C and 180 rpm. Subsequently, 5% of the culture was added into three 500 mL shake flasks containing 50 mL of improved MS medium: 0.4 g/L isoleucine, 2 mg/L VB_12_, 2 g/L β-alanine, 5 mg/L VB_1_, 48 mg/L IPTG, 50 mg/L Kan, and 1 mL/L metal salt solution, and cultured at 30°C for 48 h. The collected cell cultures were used to measure biomass and d-PA production. All data were shown as the standard deviation of three repeated averages. For DPA11A/∆*panC/*pET28a*-panB*, which was used as a recombinant strain for pantoate fermentation, an additional 50 mg/L of d-PA should be added to ensure the growth of the strains based on the d-PA culture conditions. For the d-PA fermentation in a 5 L bioreactor, the initial medium composition was consistent with that used in shake flask cultures. A 200 mL of seed culture was inoculated. The initial agitation speed was set to 400 rpm and subsequently coupled to dissolved oxygen (DO) control, with the DO concentration maintained at 10% saturation. The pH was automatically regulated at 6.8 using a 50% (vol/vol) ammonia hydroxide solution, with feeding linked to pH control. The feeding medium contained glucose (500 g/L), ammonium sulfate (10 g/L), yeast extract (2 g/L), KH_2_PO₄ (14 g/L), magnesium sulfate (8 g/L), β-alanine (40 g/L), and a trace metal solution (1 mL/L). Isoleucine (40 g/L) was supplemented intermittently.

### Analytical methods

The enzymatic activity of KPHMT was measured by GC. The reaction product, ketopantoate, undergoes lactonization under acidic conditions to form KPL, which is then quantified by GC. The ThermoFisher Scientific GC system (TRACE 1300) was equipped with the Agilent CycloSil-B capillary column (0.25 mm × 0.25 µm × 30 m, USA). A hydrogen flame ionization detector (HFID) with helium as the carrier gas was used in this study. The temperatures of the inlet and detector were adjusted to 250°C. Initially, the column temperature was set at 100°C, gradually increased to 140°C with a heating rate of 10°C/min, maintained for a duration of 5 min, and then swiftly returned back to its initial value of 100°C within 2 min. The retention time of ketopanolactone was 6.6 min.

d-PA and pantoate were quantified using high-performance liquid chromatography (HPLC). An Agilent system equipped with a C_18_ column (250 × 4.6  mm, 5 μm) was used for separation and quantification. The mobile phase was composed of water, acetonitrile, and phosphoric acid (949:50:1, vol/vol/vol) delivered at a flow rate of 0.9 mL/min under isocratic conditions. Column temperature was maintained at 40°C, and detection was carried out at 200 nm. Quantitation was based on external calibration curves prepared from authentic standards.

## Data Availability

The data that support the findings of this study are available from the corresponding author upon reasonable request.

## SUPPLEMENTAL MATERIAL

Supplemental material aem.00267-26-s0001.docx Tables S1 and S2; Fig. S1 to S4.

Graphical abstract aem.00267-26-s0002.tif Visual depiction of the study highlights.

ASM does not own the copyrights to Supplemental Material that may be linked to, or accessed through, an article. The authors have granted ASM a non-exclusive, world-wide license to publish the Supplemental Material files. Please contact the corresponding author directly for reuse.
