# Supplementary material for: Rational selection of ketopantoate hydroxymethyltransferase with low product feedback inhibition based on binding free energy calculation analysis
Source: Appl Environ Microbiol. 2026 Jul 10;92(8):e00267-26. doi: 10.1128/aem.00267-26 (PMC13488298; doi:10.1128/aem.00267-26)
Supplement: Supplementary file 1 [file aem.00267-26-s0001.docx]

**Rational selection of ketopantoate hydroxymethyltransferase with low product feedback inhibition based on binding free energy calculations analysis**

Ken Zheng^1,2,3^, Ai-Ping Pang^1,2,3^, Fang-Ying Zhu^1,2,3^, Yu-Hui Liu^1,2,3^, Xiao-Jian Zhang^1,2,3^, Qing Yang^1,2,3^, Xiang-Yang Li^1,2,3^, Jun-ping Zhou^1,2,3^, Bo Zhang^1,2,3^, Zhi-Qiang Liu^1,2,3,^*, Yu-Guo Zheng^1,2,3^

1 State Key Laboratory of Green Chemical Synthesis and Conversion, Zhejiang University of Technology, Hangzhou, Zhejiang 310014, P. R. China

2 The National and Local Joint Engineering Research Center for Biomanufacturing of Chiral Chemicals, Zhejiang University of Technology, Hangzhou, Zhejiang 310014, P. R. China

3 Key Laboratory of Bioorganic Synthesis of Zhejiang Province, Zhejiang University of Technology, Hangzhou, Zhejiang 310014, P. R. China

*Corresponding author: College of Biotechnology and Bioengineering, Zhejiang University of Technology, Hangzhou 310014, China

Tel: +86-571-88320614, Fax: +86-571-88320630, E-mail: microliu@zjut.edu.cn

**Supplementary data**

**Table S1 Gene mining sources and conserved regions.**

**Table S2 Strains, plasmids, and protein sequences were used in this study.**

**Fig. S1 The detailed KPHMT enzyme activity assay.**

**Fig. S2 Multiple sequence alignment was performed using ESPript 3.2. Key conserved residues are labeled.**

**Fig. S3 SDS‑PAGE analysis of purified KPHMT homologs. Approximately 10 μg of each purified enzyme was loaded per lane. lane 1. Crude enzyme from *E. coli* BL21 (DE3)/pET28b-*Ec*KPHMT; lane 2. Pure enzyme of *Ec*KPHMT; lane 3. Crude enzyme from *E. coli* BL21 (DE3)/pET28b-*Cg*KPHMT; lane 4. Pure enzyme of *Cg*KPHMT; lane 5. Crude enzyme from *E. coli* BL21 (DE3)/pET28b-*Bs*KPHMT; lane 6. Pure enzyme of *Bs*KPHMT; lane 7. Crude enzyme from *E. coli* BL21 (DE3)/pET28b-*Mp*KPHMT; lane 8. Pure enzyme of *Mp*KPHMT; lane 9. Crude enzyme from *E. coli* BL21 (DE3)/pET28b-*Ep*KPHMT; lane 10. Pure enzyme of *Ep*KPHMT.**

**Fig. S4 Citrate inhibited the enzymatic activity of KPHMT. The results of the experiments were tested by *Ec*KPHMT, which showed that citric acid had a severe inhibitory effect on the enzyme.**

**Table S1**

| **Entry** | **NCBI ID.** | **Source** | **Conserved region 1** | **Conserved region 2** | **Conserved region 3** | **Conserved region 4** |
| --- | --- | --- | --- | --- | --- | --- |
| 01 | WP 191638657 | *Escherichia coli* | LVGDS | HLGLTPQS | IPVIGIGAG | DGQILVMHD |
| 02 | WP 176608685 | *Enterobacter cloacae* | LVGDS | HLGLTPQS | IPVIGIGAG | DGQILVMHD |
| 03 | WP 139537005 | *Klebsiella michiganensis* | LVGDS | HLGLTPQS | IPVIGIGAG | DGQILVMHD |
| 04 | WP 188724039 | *Franconibacter daqui* | LVGDS | HLGLTPQS | IPVIGIGAG | DGQILVMHD |
| 05 | WP 088402375 | *Raoultella ornithinolytica* | LVGDS | HLGLTPQS | IPVIGIGAG | DGQILVMHD |
| 06 | WP 039897924 | *Cedecea davisae* | LVGDS | HLGLTPQS | IPVIGIGAG | DGQILVMHD |
| 07 | WP 097649895 | *Cronobacter sakazakii* | LVGDS | HLGLTPQS | IPVIGIGAG | DGQILVMHD |
| 08 | WP 174402287 | *Enterobacter hormaechei* | LVGDS | HLGLTPQS | IPVIGIGAG | DGQILVMHD |
| 09 | WP 194565313 | *Cronobacter malonaticus* | LVGDS | HLGLTPQS | IPVIGIGAG | DGQILVMHD |
| 10 | WP 002436905 | *Atlantibacter hermannii* | LVGDS | HLGLTPQS | IPVIGIGAG | DGQILVMHD |
| 11 | WP 064516274 | *Buttiauxella gaviniae* | LVGDS | HLGLTPQS | IPVIGIGAG | DGQILVMHD |
| 12 | AMX06733 | *Enterobacter asburiae* | LVGDS | HLGLTPQS | IPVIGIGAG | DGQILVMHD |
| 13 | WP 038886650 | *Cronobacter dublinensis* | LVGDS | HLGLTPQS | IPVIGIGAG | DGQILVMHD |
| 14 | WP 075194038 | *Cronobacter muytjensii* | LVGDS | HLGLTPQS | IPVIGIGAG | DGQILVMHD |
| 15 | WP 007676931 | *Cronobacter condimenti* | LVGDS | HLGLTPQS | IPVIGIGAG | DGQILVMHD |
| 16 | WP 110025785 | *Mangrovibacter plantisponsor* | LVGDS | HLGLTPQS | IPVIGIGAG | DGQILVMHD |
| 17 | WP 172913150 | *Pantoea stewartii* | LVGDS | HLGLTPQS | IPVIGIGAG | DGQILVMHD |
| 18 | WP 062747167 | *Erwinia persicina* | LVGDS | HLGLTPQS | IPTIGIGAG | DGQILVMHD |
| 19 | WP 156293078 | *Serratia oryzae* | LVGDS | HLGLTPQS | IPVIGIGAG | DGQILVMHD |
| 20 | WP 152323072 | *Erwinia endophytica* | LVGDS | HLGLTPQS | IPVIGIGAG | DGQILVMHD |
| 21 | WP 006321083 | *Serratia plymuthica* | LVGDS | HLGLTPQS | IPVIGIGAG | DGQILVMHD |
| 22 | WP 058770597 | *Pantoea dispersa* | LIGDS | HLGLTPQS | IPVIGIGAG | DGQILVMHD |
| 23 | WP 068902221 | *Enterovibrio pacificus* | LVGDS | HLGLTPQS | IPVIGIGAG | DGQILVMHD |
| 24 | WP 028864864 | *Psychromonas aquimarina* | LVGDS | HLGLTPQS | IPVIGIGAG | DGQILVMHD |
| 25 | WP 039033097 | *Shewanella* | LVGDS | HIGLTPQS | IPVIGIGAG | DGQILVMHD |
| 26 | WP 194091400 | *Vibrio hibernica* | LVGDS | HLGLTPQS | IPVIGIGAG | DGQILVMHD |
| 27 | WP 117318190 | *Salinimonas sediminis* | LIGDS | HLGLTPQS | IPVIGIGAG | DGQILVMHD |
| 28 | WP 128343799 | *Aeromonas caviae* | LIGDS | HLGLTPQS | IPVIGIGAG | DGQILVMHD |
| 29 | WP 050667912 | *Aeromonas schubertii* | LIGDS | HLGLTPQS | IPVIGIGAG | DGQILVMHD |
| 30 | WP 138622508 | *Pseudoalteromonas sp. S1610* | LVGDS | HLGLTPQS | IPTIGIGAG | DGQILVMHD |
| 31 | WP 014499355 | *Buchnera aphidicola* | LVGDS | HLGLTPQS | IPVIGIGSG | DGQILVMHD |
| 32 | WP 109113000 | *Aeromonas caviae* | LIGDS | HLGLTPQS | IPVIGIGAG | DGQILVMHD |
| 33 | WP 046357029 | *Pseudoalteromonas luteoviolacea* | LVGDS | HLGLTPQS | IPVIGIGAG | DGQILVMHD |
| 34 | WP 026376495 | *Aestuariibacter salexigens* | LIGDS | HLGLTPQS | IPVIGIGAG | DGQVLVMHD |
| 35 | WP 092840114 | *Idiomarina donghaiensis* | LVGDS | HLGLLPQS | IPVIGIGAG | DGQILVMHD |
| 36 | WP 090362331 | *Ferrimonas sediminum* | LVGDS | HLGLTPQS | IPVIGIGAG | DAQILVMHD |
| 37 | WP 126785049 | *Idiomarina seosinensis* | LVGDS | HLGLLPQS | IPTIGIGAG | DGQILVMHD |
| 38 | WP 003225584 | *Bacillus subtilis* | LVGDS | HLGLTPQS | IPVIGIGAG | DGQVLVYHD |
| 39 | WP 034982403 | *Corynebacterium glutamicum* | LVGDS | HIGYTPQS | ITTIGIGAG | DGQVLVWQD |
| 40 | WP 201516851 | *Gulosibacter hominis* | LVGDS | HVGFTPQS | IPTIGIGAG | DGQILVWQD |
| 41 | WP 066839510 | *Corynebacterium stationis* | LVGDS | HIGFTPQS | IATIGIGAG | DGQILVWQD |
| 42 | WP 284230461 | *Arenivirga flava* | LVGDS | HVGYTPQS | IPTIGIGAG | DGQILVWQD |
| 43 | WP 104296766 | *Rathayibacter toxicus* | LVGDS | HIGFTPQS | IPTIGVGAG | DGQLLVWTD |
| 44 | WP 095798245 | *Kocuria* | LVGDS | HVGFTPQS | IPTVGIGAG | TGQVLVWQD |
| 45 | WP 130980968 | *Glaciihabitans arcticus* | LVGDS | HIGYTPQS | VPTISVGAG | DGQLLVWTD |
| 46 | WP 310129778 | *Leifsonia shinshuensis* | LVGDS | HIGFTPQS | IPTVGVGAG | DGQLLVWTD |
| 47 | WP 122936083 | *Agromyces tardus* | LVGDS | HIGYTPQS | IPTISVGAG | DGQLLVWTD |
| 48 | WP 189120551 | *Nocardioides luteus* | LVGDS | HIGFTPQS | IPTIGIGAG | DGQVLVWQD |
| 49 | WP 044494848 | *Nesterenkonia massiliensis* | LVGDS | HVGFTPQS | IPTIGIGAG | TGQVLVWQD |
| 50 | WP 131385258 | *Corynebacterium callunae* | LVGDS | HIGYTPQS | IITIGIGAG | DGQVLVWQD |
| 51 | WP 191733082 | *Corynebacterium gallinarum* | LVGDS | HIGFTPQS | IATIGIGAG | DGQVLVWQD |
| 52 | WP 075664327 | *Corynebacterium frankenforstense* | LVGDS | HIGYTPQS | IPTIGIGAG | DGQILVWTD |
| 53 | WP 124969052 | *Gulosibacter macacae* | LVGDS | HVGFTPQS | IPTIGIGAG | DGQILVWTD |
| 54 | WP 256008677 | *Corynebacterium pseudogenitalium* | LVGDS | HIGFTPQS | IPTIGIGAG | DGQVLVWQD |
| 55 | WP 202852566 | *Bacillus halotolerans* | LVGDS | HLGLTPQS | IPVIGIGAG | DGQVLVYHD |
| 56 | WP 071348111 | *Bacillus amyloliquefaciens* | LVGDS | HLGLTPQS | IPVIGIGAG | DGQVLVYHD |
| 57 | WP 129091598 | *Bacillus velezensis* | LVGDS | HLGLTPQS | IPVIGIGAG | DGQVLVYHD |
| 58 | WP 268359869 | *Bacillus altitudinis* | LVGDS | HLGLTPQS | IPVIGIGAG | DGQVLVYHD |
| 59 | WP 216774183 | *Metabacillus halosaccharovorans* | LVGDS | HLGLTPQS | IPTIGIGAG | DGQVLVYHD |
| 60 | WP 242174580 | *Priestia koreensis* | LVGDS | HLGLTPQS | IPTIGIGAG | DGQVLVYHD |
| 61 | WP 181535816 | *Anoxybacillus calidus* | LVGDS | HLGLTPQS | IPTIGIGAG | DGQVLVFHD |
| 62 | WP 014460974 | *Bacillaceae* | LVGDS | HLGLTPQS | IPTIGIGAG | DGQVLVYHD |
| 63 | WP 124563904 | *Ectobacillus antri* | LVGDS | HLGLTPQS | IPTIGIGAG | DGQVLVYHD |
| 64 | WP 008879587 | *Geobacillus thermodenitrificans* | LVGDS | HLGLTPQS | IPVIGIGAG | DGQVLVYHD |
| 65 | WP 066251892 | *Aeribacillus* | LVGDS | HLGLTPQS | IPTIGIGAG | DGQVLVFHD |

**Table S2**

| Strains/plasmid | Characteristics | Source |
| --- | --- | --- |
| strains |  |  |
| *E.coli* DH5α | *E.coli* cloning strain | Tsingke |
| *E.coli* BL21 (DE3) | *E.coli* expression strain | Tsingke |
| DPA11A | *E. coli* W3110 derivative, Trc-*panC/*Trc*-panE/*Trc*-panB/*Trc-*ilvC/ilvG* /∆avtA/ilvE*/coaA*/∆ilvA/Trc-lpd/∆glk/ilvA** | Laboratory  stored |
| DPA11A/*∆panC* | *E. coli* W3110 derivative, *∆panC/*Trc*-panE/*Trc*-panB/*Trc-*ilvC/ilvG* /∆avtA/ilvE*/coaA*/∆ilvA/Trc-lpd/∆glk/ilvA** | Laboratory  stored |
| Plasmids |  |  |
| pTrc99a(K) | pTrc99a (Replace Amp^R^ with Kan^R^) | Laboratory  stored |
| pTrc99a-*Ec*KPHMT-*CgpanC* | pTrc99a(K), Kan^R^, containing *panB* from *E. coli* and *panC* from *C. glutamicum* | This study |
| pTrc99a-*Cg*KPHMT-*CgpanC* | pTrc99a(K), Kan^R^, containing *panB* and *panC* from *C. glutamicum* | This study |
| pTrc99a-*Mp*KPHMT-*CgpanC* | pTrc99a(K), Kan^R^, containing *panB* from *Mangrovibacter plantisponsor* and *panC* from *C. glutamicum* | This study |
| pTrc99a-*Ep*KPHMT-*CgpanC* | pTrc99a(K), Kan^R^, containing *panB* from *Enterovibrio pacificus* and *panC* from *C. glutamicum* | This study |
| pTrc99a-*Bs*KPHMT-*CgpanC* | pTrc99a(K), Kan^R^, containing *panB* from *Bacillus subtilis* and *panC* from *C. glutamicum* | This study |
| pET28a-*Ec*KPHMT | pET28a, containing *panB* from *E. coli* | This study |
| pET28a-*Cg*KPHMT | pET28a, containing *panB* from *C. glutamicum* | This study |
| pET28a-*Mp*KPHMT | pET28a, containing *panB* from *Mangrovibacter plantisponsor* | This study |
| pET28a-*Ep*KPHMT | pET28a, containing *panB* from *Enterovibrio pacificus* | This study |
| pET28a-*Bs*KPHMT | pET28a, containing *panB* from *Bacillus subtilis* | This study |
| Protein sequence of *Ec*KPHMT | MKPTTISLLQKYKQEKKRFATITAYDYSFAKLFADEGLNVMLVGDSLGMTVQGHDSTLPVTVADIAYHTAAVRRGAPNCLLLADLPFMAYATPEQAFENAATVMRAGANMVKIEGGEWLVETVQMLTERAVPVCGHLGLTPQSVNIFGGYKVQGRGDEAGDQLLSDALALEAAGAQLLVLECVPVELAKRITEALAIPVIGIGAGNVTDGQILVMHDAFGITGGHIPKFAKNFLAETGDIRAAVRQYMAEVESGVYPGEEHSFHHHHHHH | This study |
| Protein sequence of *Cg*KPHMT | MSGIDAKKIRTRHFREAKVNGQKVSVLTSYDALSARIFDEAGVDMLLVGDSAANVVLGRDTTLSVTLDEMIVLAKAVTIATKRALVVVDLPFGTYEVSPNQAVESAIRVMRETGAAAVKIEGGVEIAQTIRRIVDAGIPVVGHIGYTPQSEHSLGGHVVQGRGASSGKLIADARAVEQAGAFAVVLEMVPAEAAREVTEDLSITTIGIGAGNGTDGQVLVWQDAFGLNRGKKPRFVREYAALGDSLNDAAQAYIADIHAGTFPGEAESFHHHHHH | This study |
| Protein sequence of *Mp*KPHMT | MKPTTMTHLRQLKDQNKKFATITAYDFSFAKLFFDEGIRVMLVGDSLGMTVQGHDSTLPVTVADIAYHTTAVRRGAPECLLLADLPFMAYATPEQAFENAATVMRAGANMVKIEGGSWLADTVRMLTERAVPVCGHLGLTPQSVNIFGGYKVQGRDTAAAETLFDDAKALEAAGAQMLVLECVPVALAERVTQALSIPVIGIGAGNVTDGQILVMHDAFGITGGHIPKFAKDFLREAGDIRAAVRAYVHEVETGVYPGEEHSFQHHHHHH | This study |
| Protein sequence of *Ep*KPHMT | MKKVTINTLIAWKQEGRKFASVTAYDASFAQLFEQQQMPVLLVGDSLGMVLQGKNDTLSVTIDEIAYHTRSVRAGSPNSFLMADMPFMSYATPEQACQNAAKLMSAGANMVKLEGGDWLVDSVKMLTERAVPVCAHLGLTPQSVNVFGGFKVQGRDQAAAEKMVADALALEKAGAQVIVLECVPVALAEQITKSVSIPVIGIGAGNVTDGQILVMHDMFGISANYMPRFSKNYLAVAGDMREAVSRYIAEVESGEFPSPDHTFNHHHHHH | This study |
| Protein sequence of *Bs*KPHMT | MKTKLDFLKMKENEEPIVMLTAYDYPAAKLAEQAGVDMILVGDSLGMVVLGLDSTVGVTVADMIHHTKAVKRGAQNTFIVTDMPFMSYHLSKEDTLKNAAVIVQESGADALKLEGGEGVFESIRALTLGGIPVVSHLGLTPQSVGVLGGYKVQGKDEQSAKKLIEDSIKCEEAGAMMLVLECVPAELTAKIAEMLSIPVIGIGAGVKADGQVLVYHDIIGHGVERTPKFVKQYTQIDETIETAISGYVQDVRHRAFPEQKHSFQMNQTVLDGLYGGKHHHHHH | This study |
| Protein sequence of *Ls*KPHMT | MSEQSPVPSVHPTTSSMQALKRVRTRHFQAAKEEGAPFTGLTSYDMLTAQIFDEAGIDFLLVGDSAGNNVLGYDTTLPVTVDDLIPLTRAVAGAVKRAFVVADMPFGSYETGPLEALHTAVRFMKETGAHAVKLEGGRRSAEQIRRIVDAGIPVMAHIGFTPQSEHGLGGHLVQGRGQAAEDLLADAHAVEDAGAFAVVLEMVPADVAKRVTEELRIPTVGVGAGPHVDGQLLVWTDWAGFTTGRVPRFVKQYADLKTVLADAARAYKADVQGGAFPTQEHSFHHHHHH | This study |
| Protein sequence of *So*KPHMT | MKPTTVSHLRQWKQEQRKFATITAYDASFAQLFAEQGIKVMLVGDSLGMTLQGHDSTLPVTVADVAYHTRAVRRGAPACLLLADLPFMSYATPEQAFASSAELMRAGANMVKLEGGSWLYETVKMLTERAVPVCGHLGLTPQSVNVFGGYKVQGRDELAAKQLLEDAQNLELAGIQLLVLECVPAALARKVTEALSIPVIGIGAGNGTDGQILVMHDALGITGGHTPKFAKNFLAQSGDIRSAVQQYMQEVEQGTYPGEEHSFNHHHHHH | This study |
| Protein sequence of *Sp*KPHMT | MKPTTVTHLRQWKQEQRKFASLTAYDASFAKLFEEQGVKVLLVGDSLGMTLQGHDSTLPVTVADIAYHTRAVRRGAPACLLLADMPFMSYATPELAFANAAELMQAGANMVKIEGGSWLCETVKMLTERAVPVCGHLGLTPQSVNVFGGYKVQGRDELAAKQLLEDAQNLELAGMQLLVLECVPTELARKITEALSIPVIGIGAGNVTDGQILVMHDAFGITGGHTPKFAKNFLAQSGDIRLAVQQYMQEVEQGAYPAEEHSFNHHHHHH | This study |
| Protein sequence of *Cc*KPHMT | MKPTTISQLRRLKESGKKFATITAYDFSFAKLFEEEGIGVMLVGDSLGMTVQGHDSTLPVTVDDIAYHTRAVRRGAPHCLLLADLPFMAYPTPEQAFENSAAVMRAGANMVKIEGGRWLADTVRMLTERAVPVCGHLGLTPQSVNIFGGYKVQGRDEAAAQTLLDDALALEVAGAQLLVLECVPVALAQRITSALSIPVIGIGAGNVTDGQILVMHDAFGITGGHIPKFAKNFLAQAGDMRAAVREYIAEVESGVYPGAEHSFNHHHHHH | This study |
| Protein sequence of *Ae*KPHMT | MKTKLDFIQMKQKQEPIVMMTAYDYPSAKLAEQANVDMILVGDSLGMVVLGYDSTVPVTVEDMIHHTKAVKRGAKNTFVVTDMPFMSYHISTEDTLQNAKKMVQQSGADALKVEGPVFDVISLLTNAGIPVVAHLGLTPQSVGVLGGYKVQGKEAKSAEKLLLDAKKCEQAGAIAIVLECIPFQLAEKITNELSIPTIGIGAGKYTDGQVLVFHDLIGFGVERVPKFVKQYANVNESIVKAIEAYKDEVKNRDFPSLDHSFRMNEEELASLYGGKGKHHHHHH | This study |
| Protein sequence of *Ko*KPHMT | MTATPQSDQASTTPSLSEVTRVRTVHLQRAKAQGEKFAMLTSYDSLTAAIFDGAGVDVLLVGDSAGNNVLGYETTIPVTVDQMIPLAAAVARATQRALVVVDLPFGSYEESPEQAVRTSVRIMKETGAHAVKLEAGEELADHVAAVVRAGIPVMGHVGFTPQSEHALGGYRVQGKGDAAAKLIADAQAVQAAGAFAVVLEMVSADAAEEVEDILSIPTVGIGAGNSTTGQVLVWQDMLGLRTGRMPRFVKQYAQLSETIGDAVGSYVAEVKSGTFPAEEHSFRGHHHHHH | This study |
| Protein sequence of *Vh*KPHMT | MKKVSIHTLIKWKQEGHKFATSTAYDASFAQLFEQQQMPLLLVGDSLGMVLQGEDSTLPVTIDELAYHTRCVRAGNKTSLLVADMPFMTYATPDQACQEAAKLMRAGANMVKLEGGEWLIETVKQLTERSVPVCAHLGLTPQSVNIFGGFRIQGRDHDKAEQMVRDALSLQNAGAQLLVLECVPTALAERITSALHIPVIGIGAGNKTDGQILVMHDMFGISANYMPKFSKNFLAETGSMALAIDKYIQDVEAGTFPGIEHSFEHHHHHH | This study |
| Protein sequence of *Ee*KPHMT | MKPTTVSTLHQWKQQGRKFASITAYDFSFAKLFADEGIEVMLVGDSLGMVVQGHDSTLPVTVADIVYHTQAVRRGSPAALLLADLPFMSYATPEQTFDNAAQLMRAGANMVKLEGGKWLAETVRQLTERAVPVCGHLGLTPQSVNIFGGYKVQGRDSAAADRLLEDALALEAAGMQLLVLECVPVALAQRITDALTVPTIGIGAGNVTDGQILVMHDAFGITGGHIPKFAKNFLAEAGEIRAAVRQYVAEVADGRYPAAEHSFQHHHHHH | This study |

**Fig. S1**

**Fig. S2**

**Fig.S3**

**Fig. S4**
